# Proprioceptive Terrain Classification for Hexapod Robots with Statistical and Spectral Features

**DOI:** 10.3390/biomimetics11090605

**Published:** 2026-08-25

**Authors:** Deniz Korkmaz, Gonca Ozmen Koca, Cafer Bal, Mustafa Ay, Zuhtu Hakan Akpolat

**Affiliations:** 1 Department of Electrical and Electronics Engineering, Faculty of Engineering and Natural Sciences, Malatya Turgut Ozal University, Malatya 44900, Turkey; 2 Department of Mechatronics Engineering, Faculty of Technology, Firat University, Elazig 23119, Turkey; gozmen@firat.edu.tr (G.O.K.); cbal@firat.edu.tr (C.B.); mustafaay@firat.edu.tr (M.A.); 3 Department of Electrical and Electronics Engineering, Faculty of Engineering, Fatih Sultan Mehmet Vakif University, Istanbul 34015, Turkey; zhakpolat@fsm.edu.tr

**Keywords:** hexapod robot, terrain classification, feature extraction, inertial measurement unit, proprioceptive sensing

## Abstract

Terrain types significantly affect the dynamics and locomotion performance of hexapod robots during walking. Terrain classification is a key solution to modify gait patterns in different terrains and recognize hazardous conditions. Perceiving the terrain with proprioceptive sensing is a robust and reliable approach in extreme conditions. In this paper, an efficient terrain classification approach for a hexapod robot is proposed. The proposed method combines a deep classification framework including the long short-term memory (LSTM) network and an effective statistical feature extraction. Proprioceptive inertial measurement unit (IMU) data is only used as the sensing system for the robot–terrain interaction. In the feature extraction process, four meaningful characteristic features, namely, the mean, median, Lomb–Scargle periodogram power spectral density (LPSD), and Welch’s power spectral density (WPSD), are extracted from the body orientation data using a sliding-window method. These features are combined and fed into the network to perform the training and testing processes. In the experiments, the proposed method is evaluated with commonly used soft computing and deep learning models. The classification performance for the concrete, pebble, and waxed tile terrains reaches 100% with the proposed method. The overall accuracy, precision, sensitivity, specificity, F1-score, and Matthew correlation coefficient are recorded as 95.45%, 96.36%, 95.28%, 98.86%, 95.49%, and 94.61%, respectively. These results demonstrate that the proposed approach delivers reliable classification performance with a low-cost and easy-to-implement solution.

## 1. Introduction

In recent years, autonomous robots have been frequently used in many tasks such as surveillance, inspection, rescue, and exploration operations, where human actions are limited or dangerous. Since autonomous robots must operate in unpredictable scenarios, mission success depends on their ability to autonomously adapt their motion to the terrain topology, environmental conditions, and obstacles [1,2]. Among the locomotion types, multi-legged robots have attracted increasing interest for tedious, laborious, and dirty tasks such as routine inspection and monitoring, owing to their ability to traverse complex, unstructured terrain and overcome obstacles by applying only reaction forces to the ground [3,4]. It is possible to adjust the behavior of multi-legged robots and overcome the challenges in their environment by changing their gaits, foot tip shapes, leg placements, stride lengths, etc. Since multi-legged robots are designed as over-actuated, multi-body, and rigid-flexible systems with complex interactions in the environment, they have excellent flexibility to develop hardware and software configurations more appropriate to the specific characteristics of the terrain [5,6]. According to various configurations, the leg mechanism can be used as a robot arm, and unlike wheeled or tracked robots tipping over on inclined terrain, multi-legged robots can maintain their balance with their legs. In addition, the main body of the legged robots can carry out machining or maintenance tasks with mounted tools on the body, considering a mobile platform [7,8].

Legged robots represent an important field of biomimetics because their mechanical structures and locomotion strategies are inspired by the morphology and movement principles of animals and insects. In particular, hexapod robots imitate the multi-legged organization of insects, enabling stable locomotion and flexible gait generation on complex terrains. Biological locomotion principles can also be reproduced through central pattern generators (CPGs), which mimic neural mechanisms responsible for generating rhythmic motor patterns in animals. Although multi-legged robots have the ability to move in complex environments with their natural stability, the structure of the terrain surface influences the locomotion performance during interaction with the environment. Properties such as the coefficient of friction, compliance, and shape of the part on the ground have an impact on walking stability and determine the overall reliability of the posture [9,10]. On flat terrain, the robot can perform a periodic gait to move smoothly. However, on complex terrain, the robot needs to determine gait planning and the information about the type of terrain allows the robot to adapt to changing conditions using appropriate gait patterns and to provide stable and efficient motion control. In particular, granular terrains with different fractions significantly affect the safety of locomotion and efficient energy consumption. While low friction may cause slipping, unconformity may prevent the body displacement with vibration. Therefore, the classification of different terrains is required to control their gaits in achieving adaptive autonomous behavior [11]. The ability to accurately perceive and classify the terrain allows the robot to perform accurate gait planning, path planning, and motion control strategies [12,13]. Efficient locomotion on complex terrain assists in minimizing energy costs during walking [14]. Advances in artificial intelligence and machine learning have led to significant progress in this field. Compared to conventional methods, deep learning can achieve superior classification accuracy with effective feature extraction and high generalization capability. Therefore, multi-legged robots could identify the terrain with good accuracy using sensors installed on their legs or bodies and perform given tasks while walking on uneven terrains. As a result, terrain perception is essential for enabling a legged robot to adapt its locomotion according to environmental conditions, similar to the way biological insects modify their motion when interacting with different surfaces.

### 1.1. Related Works

In recent years, the topic of terrain classification has started to gain attention and has been investigated for a wide variety of autonomous robots, including wheeled [15], crawler [16], biped [17], exoskeletons [18], and multi-legged robots [19,20]. The applications generally consist of using sensor modalities to gather information about the terrains from robots and designing an efficient classifier model to predict a terrain label during operation. Exteroceptive and proprioceptive sensing can address legged robot locomotion in uneven and unstructured terrains. Among them, exteroceptive sensors, such as vision, depth, and lidar, allow a robot to determine its environment without direct interaction [21,22]. The proprioceptive sensing-based methods define the terrain through the interaction between the robot and the environment using various sensors such as joint, acoustic, tactile, force, and inertial measurement units (IMUs) [23,24].

Numerous studies have been conducted on terrain classification under various conditions and scenarios, particularly for wheeled robots. Chen et al. [25] proposed a terrain classification framework for a ground robot that combines visual and proprioceptive sensing under low-light conditions. A zero-order hold (ZOH) interpolation was employed to determine the sampling frequency, and a fuzzy logic model was utilized to tune the equalization. Fritz et al. [26] designed a classification of several off-road terrains based on road roughness. Their method utilized a convolutional neural network (CNN) model. Two cameras, with 6 mm focal length lenses, were mounted on a test vehicle. The first camera was used as a downward-facing camera and the second camera was used for forward-facing. Wang et al. [27] proposed an identification model for a wheeled robot based on the support vector machine (SVM) by using the data of oil pressure and displacement of active suspensions. Singh et al. [28] introduced a CNN-based approach to classify the surface of various terrains, such as carpet, tiles, concrete, etc., for a tiny mobile robot. The dataset was captured by the IMU, and their method achieved an overall classification accuracy of 88%. In another study, Mao et al. [29] introduced a lightweight CNN model based on an augmentation method for road surface classification under shadow situations. For different road types, an overall classification accuracy was achieved as 94.75%. Shen et al. [30] presented a visual end-to-end classification and segmentation network for a wheeled robot in the Mars Utopia Planitia region. The classification accuracy of their model achieved 83.90% and the segmentation accuracy exceeded 80%. Guan et al. [31] proposed a linear identification framework for a spherical robot with nonlinear and time-varying dynamics. Using Koopman theory, a linear model was constructed, and a Kalman filter was used for the noise estimation. The long-term prediction was performed using the collected IMU data.

Compared to wheeled robots, terrain classification studies for multi-legged robots are relatively limited. The high complexity of multi-joint mechanisms causes challenges for proprioceptive-based terrain recognition. Consequently, using visual perception has become a popular solution for detecting irregular terrain, and researchers have conducted several studies using computer vision techniques. Zhao et al. [32] proposed a deep CNN model based on the generative adversarial network (GAN) and Dense network. The onboard camera of the hexapod robot was employed to collect terrain images from unstructured environments. In their experiments, the flat land, grass, rugged ground, and various object imagery were captured. Luo et al. [33] presented an adaptive gait control approach of a quadruped robot and combined terrain recognition considering the cost of transport (CoT), and the Froude (Fr) number. EfficientNetv2 was selected as the network backbone, and feature extraction and encoding of terrain textures from images were used. Ten terrain types, including asphalt, brick, shale, and soil, were distinguished by their method. The overall classification performance was achieved in terms of accuracy, precision, and recall, which were 90.8%, 93.3%, and 90.8%. However, the prediction results still need to be improved, although their method gave a lower computational complexity. Shi et al. [34] introduced a vision-based foot mechanism for quadruped robots. Their design consisted of a compliant ankle, a transparent foot sole with markers, and an internal camera. In the experiments, grass, soil, and gravel terrains were used. The coefficient of determination was obtained as 0.95 for each force axis. Although they achieved a low-cost and adaptable mechanism for both deformable and rigid terrains, the terrain types are limited. Zhu et al. [35] proposed an adaptive locomotion method in another study for terrain classification based on a combination of speeded-up robust features and binary robust invariant scalable key points. The image infilling method was used to classify terrain types. All extracted features were processed by a clustering algorithm for six different outdoor terrain samples and the average recognition accuracy reached 85%. This method makes significant contributions to detecting obstacles during classification, but there is a need to increase the accuracy of classification performance.

Although visual perception-based methods have achieved good classification results, these approaches have some limitations. Visual-based classification can be affected in field settings due to lighting variations, shadows, dust/fog, wetness, and viewpoint effects. In these methods, although the geometry of the surface can be fixed for various terrain types, the characteristics of the terrain type may not be provided because the geometry may change during locomotion. Moreover, visual perception describes the surface texture, whereas proprioceptive sensing directly reflects the robot–terrain interaction dynamics. Remote sensing is also troublesome and has excessive design costs to completely detect the physical information of the environment [11,24].

Compared to visual classification, proprioceptive sensing is less affected by lighting changes and reduced visibility, while also reducing computational and data requirements. In proprioceptive sensing, the number and type of force, contact, and position sensors used in the literature range from single-modality and single sensor location to fusing measurements from multiple sensors in different body or leg parts. The selection and placement of the sensor type often depend on the compatibility with the robotic system and its use in classification algorithms [36]. Information about the ground is gathered with these sensor types. Recently, many different types of sensors have been investigated for indoor versus outdoor terrain problems of multi-legged robots. Lokhande et al. [37] designed a situation-driven multi-modal terrain classification architecture to provide adaptive gait control. Using the IMU data, a position and attitude estimation was built. Haddeler et al. [38] presented a ground perception model for a quadruped robot with a digital double framework for elevated, inclined, and outdoor ground types. The body orientation angle, joint angle position, and velocity were used as the input for their network. Zhang et al. [23] proposed a multi-sensor signal integration method for a quadruped robot to detect terrain types independently of the executed motion. The IMU and joint encoder measurements were used as the sensor data that defined as the body angular position and angular velocity data around the horizontal axes, and the linear velocity of the foot. The distinctly dominant features were used and labeled as four categories as roughness, slippage, softness, and slope. They achieved a high accuracy performance of 95.02%. However, more complex terrains with coupled multi-labels were not explored. Similarly, Ding et al. [11] used physics-informed and tidy-interpretable features to facilitate the haptic terrain classification of a hexapod robot. The 6-axis force sensors were installed at the connection between the foot and legs. Experiments were conducted on multi-class mixed terrains, and high classification performance was achieved using an SVM, with an accuracy of 97.50% across 10 terrain types and 85.83% across 12 terrain types. Although these studies achieved high improvements with their proposed method, the force sensors on the robot were located in the foot tips, which may cause damage to these sensors in uneven environmental conditions.

In another study, Qin et al. [39] proposed an acoustics-based online terrain classification approach for quadruped robots. The time-series data was collected with a microphone placed on the leg, sampled at 48 kHz. An efficient feature extraction process was performed and the Gaussian mixture model was used as a classifier. Using acoustic sensing, they achieved an accuracy of 92.70%. Although they obtained high classification performance, the results were presented for only indoor applications. Elnoor et al. [20] used force and current sensors to measure the joint positions, forces, and current consumption of a quadruped robot for terrain traversability estimation in a variety of environments as rocky, granular, grass, and densely vegetation. In the experiments, the principal component analysis (PCA) was used and achieved an overall success rate of 87.50%. They obtained effective estimation results for extreme outdoor terrains, but it is seen that the terrain types need to be diversified. Grezmak et al. [36] presented a hexapod terrain classification approach with passively compliant components. Terrain information was gathered by measuring low-cost hall effect magnetometers and embedded magnets. Datasets of sensor measurements were collected from three types of granular terrains in both a laboratory and outdoor beach environment and used to train SVM classifiers. Using time-domain features of measurements from each leg, an average accuracy of 92.40% through 10-fold cross-validation was achieved for the beach dataset and an average accuracy of 91.6% was obtained from onboard IMU measurements. Their method was examined for three terrain classes and the obtained results were increased by using the IMU sensor together with the magnetometer. Hu [40] introduced a terrain classification framework integrating the Fourier transform, adaptive filtering, and a deep network. While a light-weighted CNN model was used for feature extraction, a long short-term memory (LSTM) was used for classification. The data was sampled with an IMU sensor from nine distinct types of surfaces, and 81% of classification accuracy was achieved. Unlike sensing sensors, Li et al. [41] designed a rough terrain perception method for a hexapod robot using foot locomotion analysis based on the kinematic and configuration characteristics. The common support foot obtained the body posture transformation relationship in the same coordinate system. However, the experiments were performed with only artificial terrain scenarios in a laboratory. Beyond terrain classification itself, recent studies have highlighted how terrain perception can be tightly coupled with adaptive gait planning and control. Terrain perception and stiffness adaptation-based attitude control has been proposed to stabilize quadruped locomotion on varying grounds [42], and terrain recognition combined with the Froude number has been used for stable and efficient adaptive gait planning [33]. In addition, the design, modeling, and control challenges of legged robot limb units have been comprehensively discussed in [43]. These studies emphasize that reliable terrain recognition constitutes the perception module of adaptive locomotion control, which further motivates the low-cost proprioceptive classification approach proposed in this paper.

Despite the terrain classification and recognition studies, existing approaches for legged robots generally depend on additional sensing modalities such as foot/contact forces, joint torques, encoders, or vision, and many studies evaluate limited terrains. For the existing proprioceptive sensing-based studies, there are still some open issues. Firstly, the contact models between feet and different terrains may cause a lack of tactile perception. Secondly, as the sensors are required to be waterproof and dustproof, the proprioceptive sensors may be unavailable in dirty or wet environments and require special design structures. Some contact-based sensors may also be difficult to use, depending on the foot tip design. Lastly, the increasing number of sensors means high computational costs and may prevent the use of cheaper and lighter robotic designs. As a result, it can be seen that the studies on terrain classification problems for multi-legged robots need further research, and low-cost and appropriate approaches are required for uncertain environmental conditions. Among proprioceptive classification methods, IMU-based perception can avoid additional wiring, mechanical integration, and durability issues from repeated impacts, compared to contact sensors. This design can also provide a lightweight and low-cost solution suitable for embedded systems. Combining statistical and spectral features with body orientation signals and integrating this structure with a temporal network can effectively separate frequency-dependent body oscillations and amplitude-dependent variations, in contrast to typical end-to-end deep learning approaches that rely on raw signal inputs.

### 1.2. Contributions

To address the aforementioned limitations, a deep classification approach that effectively distinguishes different terrains is proposed. For sensing the robot–terrain interaction, a 9-axis IMU (with a triaxial accelerometer, gyroscope, and magnetometer) is utilized as the proprioceptive sensing modality. Unlike traditional terrain perception methods, the proposed approach utilizes the Euler measurements of the hexapod robot as the primary source of information. These measurements are inherently available in multi-legged robotic systems, thereby eliminating the need for additional external contact sensors. The proposed method intends to develop a novel deep classification framework including the LSTM network. The designed network provides a feedback loop capability that acts as a kind of memory. Therefore, the interaction of the robot in different environments is recognized as temporal patterns from dynamic data. To extract more informative representations from the IMU measurements, the four characteristic features, namely, mean (*m*), median (*med*), Lomb–Scargle periodogram power spectral density (LPSD), and Welch’s power spectral density (WPSD) are obtained from yaw, pitch, and roll angles via sliding-window method and the extracted features are used as the network input for identifying terrain types. In the robot locomotion, a CPG network-based control architecture is designed with a leg trajectory generator. The coupled CPG network behaves as an artificial spinal cord model that generates rhythmic and oscillatory gait patterns depending on the terrain. According to the design scheme, this paper aims at a high-performance terrain classification approach with a low-weight and low-cost system that makes it easily applicable in real-time operations. The proposed approach does not require external sensors (e.g., LiDAR, force sensors) by utilizing a single body-mounted IMU sensor. Furthermore, the feature-based architecture minimizes computational complexity, allowing for real-time operation on standard embedded microcontrollers with low memory usage. In the experiments, the proposed method is evaluated for commonly encountered and harsh terrains, and the obtained results are compared with the effective classification algorithms. Overall, the novelty and main contributions of this paper can be summarized as follows:

- A novel proprioceptive terrain classification system is proposed for a hexapod robot to achieve effective and high performance. The proposed method is a combination of powerful feature extraction and a time-based recurrent neural network (RNN) that provides temporal feature patterns from the angular positions.
- The designed LSTM model learns temporal terrain patterns directly from the windowed statistical and spectral feature sequences, without requiring a separate handcrafted descriptor or an additional external classifier stage. Using LPSD and WPSD provides complementary spectral information of the same windowed walking dynamics, while mean and median capture amplitude-tendency behavior. Their combination yields a richer representation for temporal learning.
- Using an IMU sensor mounted on the body, the kinematics of the robot can be extracted, which gives terrain interactions between the leg and the ground. Therefore, an effective classification approach is developed that offers high applicability, low cost, and low computation complexity.
- The proposed method provides significant knowledge about the characteristics of terrains necessary for the robot to automatically adapt its gait patterns according to the different terrain types.

The other sections of this paper are organized as follows: Section 2 describes the background theories of the proposed method. The experiments and findings of the study are given in Section 3. Finally, the conclusion of the study is briefed in Section 4.

## 2. Methodology

Accurate gait adaptability for safe locomotion in an unknown environment is considered a challenging topic for hexapod robots in a real-world scenario. During locomotion, knowledge about the terrain type is required to traverse from different grounds. Therefore, this paper is motivated to perform efficient terrain classification of a hexapod robot. The developed model aims to improve autonomous locomotion performance by adapting the variations of gaits during navigation in terrains. The following subsections present the hexapod robot prototype, CPG-based locomotion control strategy, description of the LSTM network, feature extraction process, data collection, and framework of the proposed method in detail.

### 2.1. Hexapod Robot Prototype

**Figure 1:**
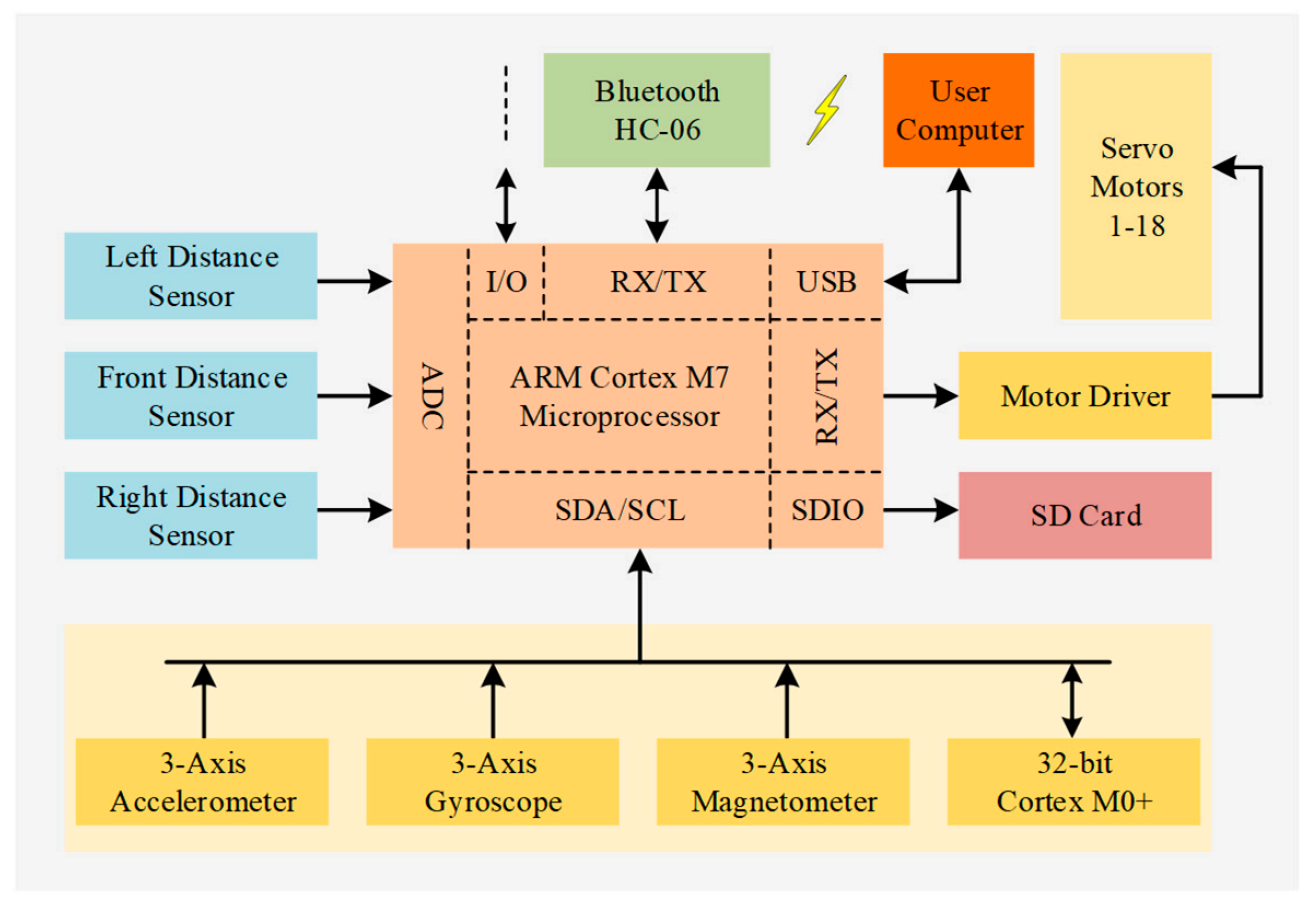
Electronic system of the hexapod robot. The Cortex-M0+ core shown in the figure is the microcontroller embedded in the IMU module, which performs the on-chip sensor fusion; the main processing unit of the robot is the ARM Cortex-M7.

**Figure 2:**
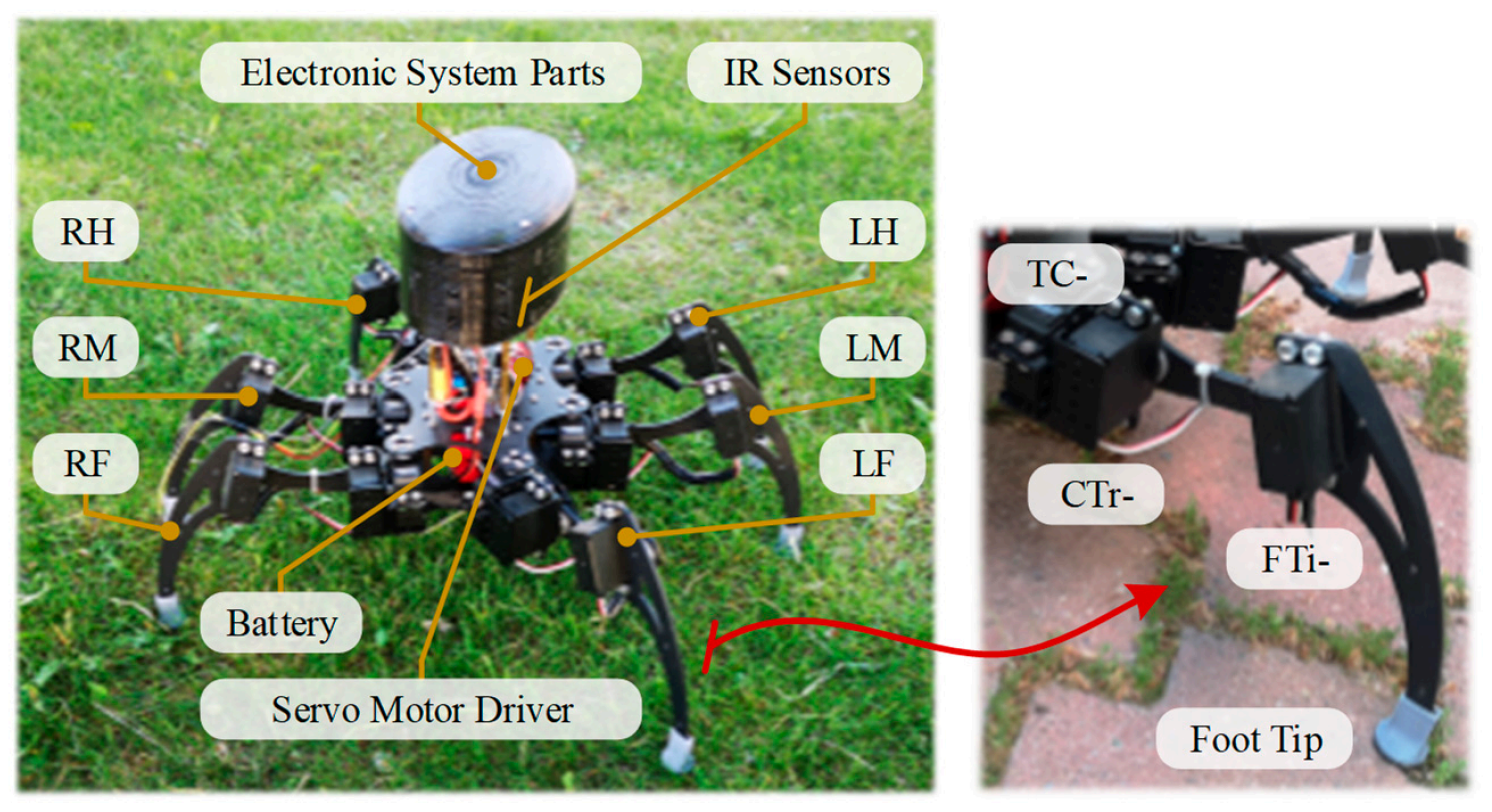
Structure of the hexapod robot prototype.

An updated version of the hexapod robot previously presented in [44] is used to collect the terrain classification data and it performs the CPG-based locomotion control method on these terrains. The robot prototype is biologically inspired by six-legged insects. Each leg has a 3-DoF structure and consists of three joints as thoracal-coxal (TC-), coxal-trochanteral (CTr-), and femoral-tibial (FTi-). TC- provides forward (+) and backward (−) motion while CTr- performs elevation (+) and depression (−) of the leg. FTi- also enables extension (+) and flexion (−) of the tibia link. Each joint of a leg is a rotary DoF driven by 2.84 Nm and 7.4 V powerful Radio Control (RC) servo motor, and each link is relative to the servo motor rotation center. While the LF, LM, and LH define the front, middle, and hind legs of the left side, the RF, RM, and RH define the front, middle, and hind legs of the right side. The length and weight of each leg are about 240 mm and 0.6 kg, respectively (i.e., total of the coxa, femur, and tibia links). The main body of the prototype consists of two rigid parts as lower and upper parts. The lower body is made of carbon fiber material and has a hexagonal shape. The diagonal sides are 85 mm and the left and right sides are 65 mm. The lower body part includes the leg mechanisms, a 20-channel servo motor driving unit, and a battery pack. In the rigid upper part, there is a microcontroller unit, environmental sensors, and an HC06 Bluetooth module. The Bluetooth module provides wireless communication between the robot and the user computer. To perceive and avoid environmental obstacles, GP2Y0A21 infrared (IR) distance sensors are used. These sensors are located horizontally parallel to each other in the front axis of the robot and the positions are centered in the same direction at angular intervals of 45°. The IMU with a triaxial 14-bit accelerometer, an accurate closed-loop triaxial 16-bit gyroscope, a triaxial geomagnetic sensor, and a 32-bit microcontroller measures the three-dimensional (3D) posture of the robot (or slope of the body) at any instance. The communication of the IMU sensor is realized through the I^2^C protocol. The locomotion control is performed by a 32-bit ARM Cortex-M7 microcontroller (NXP MIMXRT1062) operating at 600 MHz. Figure 1 presents the electronic system of the hexapod robot. All of the components are located considering the body shape and static balance of the robot. The hexapod robot can stand with a maximum height of 240 mm and a minimum height of 200 mm. The operation time and total weight of the robot prototype are nearly 30 min and 2.3 kg, respectively. One of the key advantages of the hexapod design is that, compared to quadruped robots, the hexapod robot can perform a variety of biologically inspired gaits and exceed various requirements such as leg failure. Figure 2 shows the hexapod robot prototype.

### 2.2. CPG-Based Locomotion Control Strategy

CPGs are a group of neural oscillators that generate rhythmic, stable, adaptable, and rich dynamic output activities. These neural circuit models are well-suited to the locomotion control of the hexapod robot and smooth gait transitions can be easily generated according to environmental changes [44,45]. The constructed CPG network is based on the coupled Hopf oscillator model that produces rhythmic and adaptive motor behaviors. These motor patterns enable the robot not only to initiate different higher-order behaviors such as climbing or turning, but also to locally adapt its leg and postural control. The coupled Hopf oscillator is represented by the following equations:

(1) $$\dot{x}=\alpha \left(\mu -r^{2}\right)\left(x-u_{1}\right)-\sigma \left(y-u_{2}\right)+\varepsilon_{ij}\Gamma_{ij}$$

(2) $$\dot{y}=\alpha \left(\mu -r^{2}\right)\left(y-u_{2}\right)+\sigma \left(x-u_{1}\right)+\varepsilon_{ij}\Gamma_{ij}$$

where x and y are the state variables of the oscillator outputs, $\left(u_{1},u_{2}\right)$ are the external feedbacks, and α is the positive convergence speed. $\sqrt{\mu }$ is the output amplitude and r defines the radius of the limit cycle with $r^{2}={\left(x-u_{1}\right)}^{2}+{\left(y-u_{2}\right)}^{2}$. In the oscillator, r is decreased if $r^{2}>\mu$ and $\dot{r}<0$. If $r^{2}<\mu$ and $\dot{r}>0$, r is increased. Therefore, r is a fixed point at $\sqrt{\mu }$. The natural oscillator frequency is symbolized as σ and expressed by the following:

(3) $$\sigma =\frac{\pi }{T\beta \left(e^{-by}+1\right)}+\frac{\pi }{T\left(1-\beta \right)\left(e^{by}+1\right)}$$

where T is the output period, β is the duty factor, and b is the positive transformation constant. For the *j*th oscillator of the network, the coupling is expressed as follows:

(4) $$\Gamma_{ij}=\omega_{ij}\sum_{i\neq j}^{N}R\left(\phi_{ij}\right)f\left(x_{j},y_{j}\right)$$

Here, j=1,2,…,N is the oscillator number and N is the total oscillator number for j≠i. $R\left(\phi_{ij}\right)$ indicates the two-dimensional rotation matrix with phase differences $\left(\phi_{ij}\right)$ of the *i*th and *j*th oscillators for the *j*th oscillator outputs $f\left(x_{j},y_{j}\right)$. The trajectory generator strategy aims to achieve the desired walking gait and speed with the CPG network outputs. For a leg motion in one period, two phases are defined as swing and stance. While the robot walks at a constant speed, *i*th leg tip position of the trajectory generator $\left(P_{y,i}\right)$ can be presented as $\left[\begin{array}{ccc} P_{y,i}\left(0\right) & P_{y,i}\left(T\right) & P_{y,i}\left(2T\right) \end{array}\right]=\left[\begin{array}{ccc} PEP & AEP & PEP \end{array}\right]$. Here, PEP and AEP are the starting and finishing positions of the leg in the forward motion. In addition, PEP is Ω/2 and AEP is −Ω/2. Here, Ω represents the stance distance of the leg tip. For the reference swing trajectory vector $P_{i}^{sw}={\left[\begin{array}{ccc} P_{x,i}^{sw} & P_{y,i}^{sw} & P_{z,i}^{sw} \end{array}\right]}^{T}$, the motion of the tip starts at the time $t_{i}^{sw}$ and ascends to maximum height H and finishes at the time $t_{i}^{st}$. According to this definition, the reference trajectory of a leg for the swing phase can be generated as follows:

(5) $$\left[\begin{array}{c} P_{x,i}^{sw}\\ P_{y,i}^{sw}\\ P_{z,i}^{sw} \end{array}\right]=\left[\begin{array}{c} 0\\ \frac{\upsilon T_{i}^{sw}}{2}+\upsilon \left(t-t_{i}^{sw}\left(k\right)\right)-\upsilon T_{i}^{sw}\cos\left(\frac{\pi }{T_{i}^{sw}+T_{i}^{st}}\left(t-t_{i}^{sw}\left(k\right)\right)\right)\\ H_{i}\left(x_{i}-\nabla \right)/\left(1-\nabla \right) \end{array}\right]$$

Here, υ is the forward speed and ∇ is the threshold of the CPG outputs; $T^{st}$ and $T^{sw}$ are the stance and swing periods of a leg, respectively. Therefore, $T_{st}+T_{sw}$ gives the total gait cycle of a leg. This cycle is also repeated for the reference trajectory of the stance phase $P_{i}^{st}={\left[\begin{array}{ccc} P_{x,i}^{st} & P_{y,i}^{st} & P_{z,i}^{st} \end{array}\right]}^{T}$ with starting at the time $t_{i}^{st}$ and ending at the time $t_{i}^{sw}$ as below:

(6) $$\left[\begin{array}{c} P_{x,i}^{st}\\ P_{y,i}^{st}\\ P_{z,i}^{st} \end{array}\right]=\left[\begin{array}{c} 0\\ y_{AEP}-\upsilon \left(t-t_{i}^{st}\left(k\right)\right)\\ 0 \end{array}\right]$$

Finally, inverse kinematics of the trajectories can be easily calculated to obtain the joint angles with the aforementioned reference trajectory generator. Considering the *i*th leg, each joint angle can be derived with the following equation:

(7) $$\left[\begin{array}{c} \theta_{1,i}\\ \theta_{2,i}\\ \theta_{3,i} \end{array}\right]=\left[\begin{array}{c} arctan\left(P_{y,i}/\left(P_{x}\left(0\right)+P_{x,i}\right)\right)\\ arcsin\left(\begin{array}{c} P_{z,i}/\sqrt{{\left(L_{3,i}cos\theta_{3,i}\right)}^{2}+{\left(L_{3,i}sin\theta_{3,i}\right)}^{2}}-\\ arctan\left(L_{3,i}sin\theta_{3,i}/\left(L_{3,i}cos\theta_{3,i}+L_{2,i}\right)\right) \end{array}\right)\\ arcsin\left(\begin{array}{c} {\left(\left(P_{x}\left(0\right)+P_{x,i}\right)cos\theta_{1,i}+P_{y,i}sin\theta_{1,i}-L_{1,i}\right)}^{2}+\\ {P_{z,i}}^{2}-{L_{3,i}}^{2}-{L_{2,i}}^{2} \end{array}/2L_{2,i}L_{3,i}\right)-\frac{\pi }{2} \end{array}\right]$$

**Figure 3:**
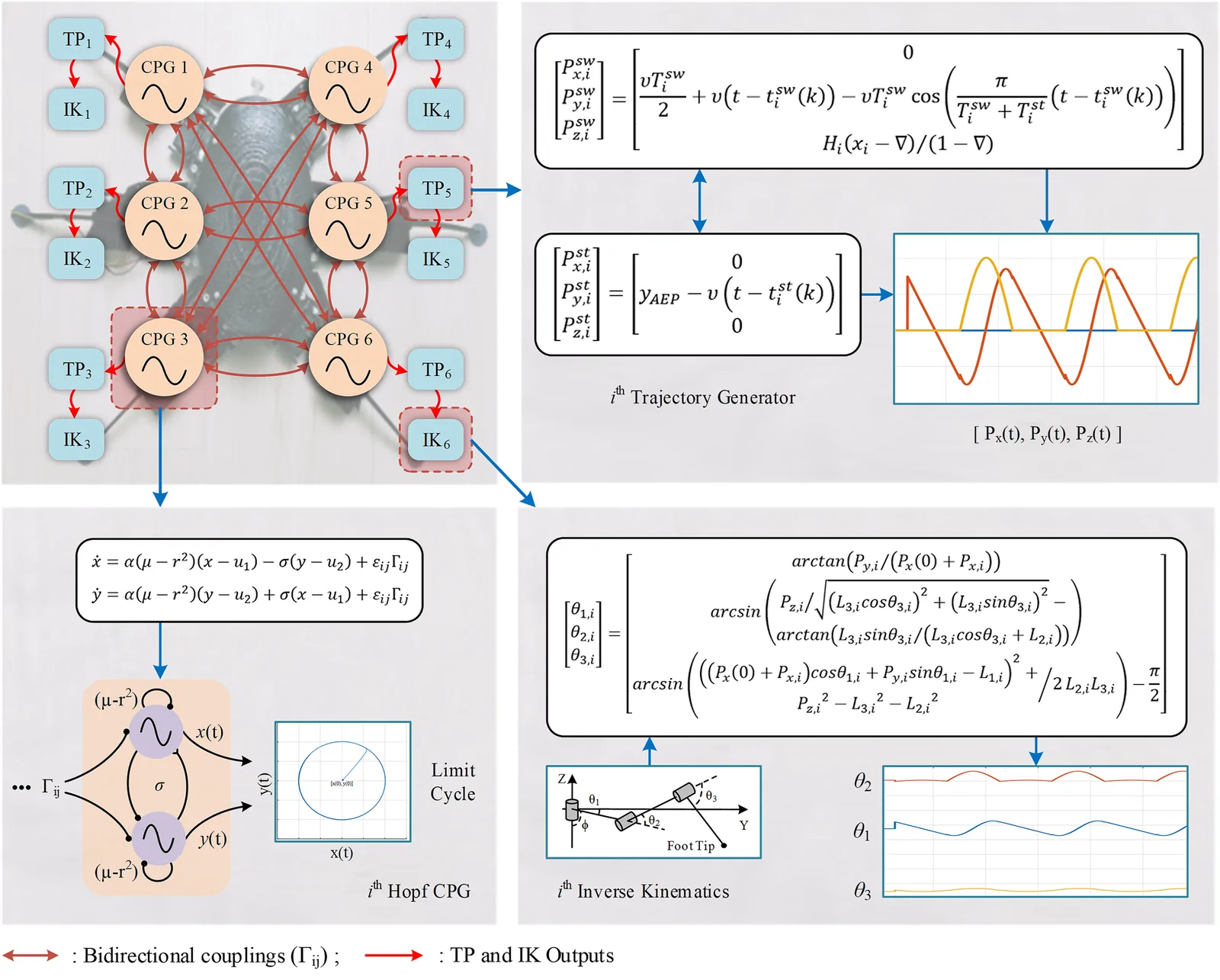
Schematic diagram of the CPG-based locomotion control. Here, TP and IK represent the trajectory generator and inverse kinematics models.

Here, $\left(L_{1},\;L_{2},\;L_{3}\right)$ define the lengths of the coxa, femur, and tibia links. As a result, the overall locomotion control structure is obtained. The whole control scheme, including the constructed CPG network, trajectory generator, and inverse kinematics, is presented in Figure 3. For the *i*th leg, the CPG output produces the reference signal for the trajectory generator. With this signal, a three-dimensional trajectory of the foot tip, including the swing and stance phases, is obtained. The output of the trajectory generator is then fed to the inverse kinematics model and the angles of the relevant joints are derived.

### 2.3. LSTM Network

**Figure 4:**
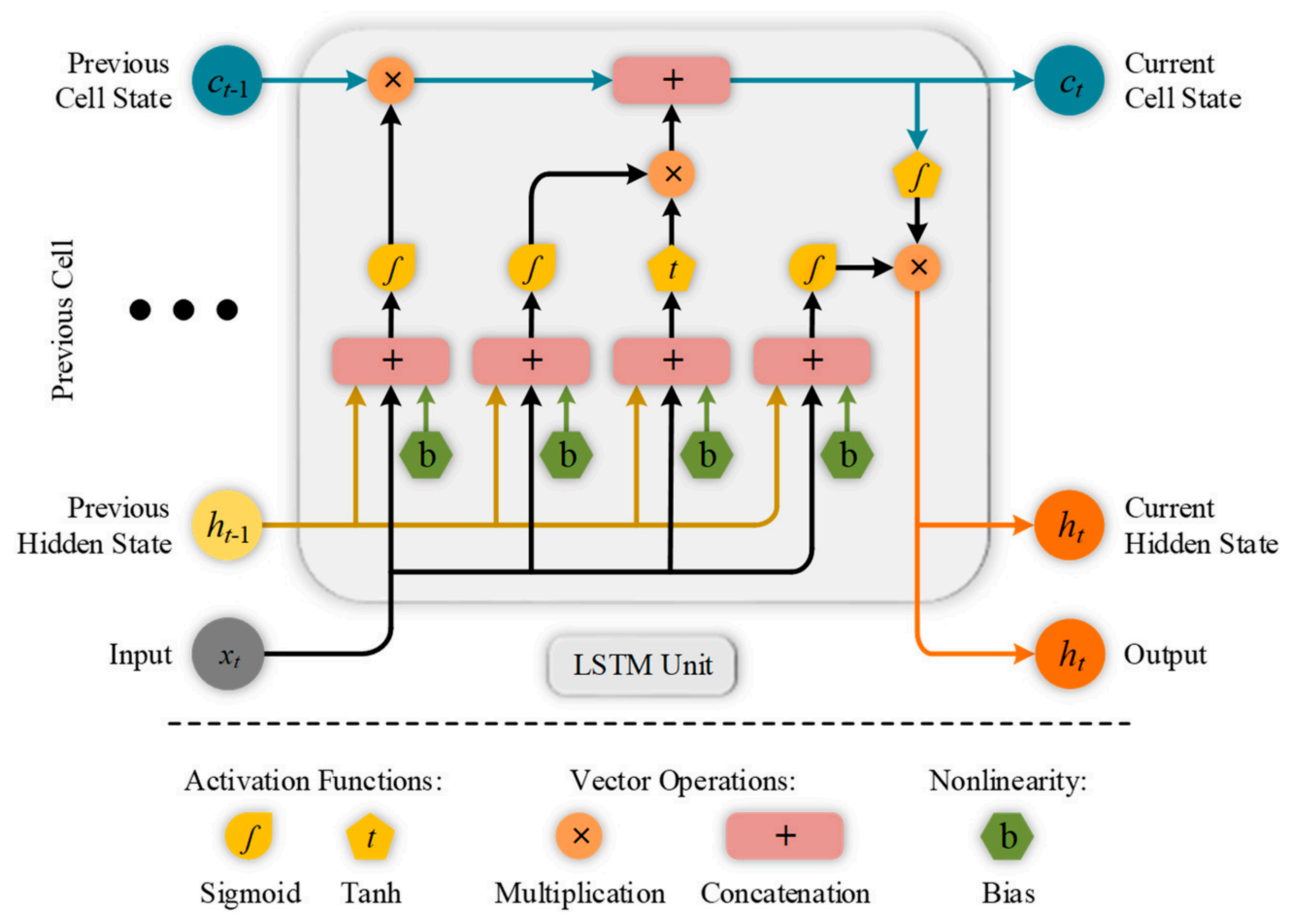
General structure of the LSTM unit.

RNN is a deep learning network that utilizes historical data to improve the performance of the network on current and future inputs. A major problem with RNNs is that the vanishing gradient problem may occur during backpropagation. LSTM network is a time-series RNN that uses internal memory loops and input data arrays to address this problem by integrating gating functions into state dynamics. Therefore, the LSTM network provides powerful processing of nonlinear sequential data to overcome exploding and disappearing gradient problems [46,47]. As shown in Figure 4, a general LSTM structure consists of an input, hidden, and output layers. The hidden layer utilizes memory cells and gate mechanisms, including a forget gate, an input gate, and an output gate. These gates keep the values determined in the previous time and control the data transmission in the units. The memory cell utilizes the product and matrix addition operations to update the hidden layers. The forget gate determines the information to be discarded from the previous cell. The input gate is responsible for processing the current state whether to update it or not. Information from the current input and hidden state is applied to the activation function to determine the degree of the information. Finally, the output gate produces the final output state [48]. Through these structural properties, LSTM has a superior ability to recognize time-based information in data. Depending on the terrain interaction between the legs and the ground in the hexapod robot, the IMU sensor gives the angular positions in the three-dimensional space. These angles are well suited for the LSTM network to construct the data classification, processing, and time-series prediction framework. The critical time-series features from IMU data are appropriately considered in both the short and long term. In the LSTM unit, the forget gate resembles a simple single-layer neural network and the activation of this gate is expressed as follows:

(8) $$f_{t}=\sigma \left(W\left[x_{t},h_{t-1},C_{t-1}\right]+b_{f}\right)$$

Here, $C_{t-1}$ and $h_{t-1}$ represent the previous cell memory and cell output; $x_{t}$ is the input sequence; σ is the sigmoid function; W and $b_{f}$ are the weight matrix and bias terms, respectively. The input gate is a part that creates the new memory by a single-layer neural network with a $tanh\;\left(\cdot \right)$ activation function and a previous memory block effect. These operations are obtained as Equations (9) and (10):

(9) $$i_{t}=\sigma \left(W\left[x_{t},h_{t-1},C_{t-1}\right]+b_{i}\right)$$

(10) $$C_{t}=f_{t}\times C_{t-1}+i_{t}\times tanh\left(W\left[x_{t},h_{t-1},C_{t-1}\right]+b_{c}\right)$$

where $b_{i}$ and $b_{c}$ are the bias terms of the input layer and cell state. Therefore, the update structure updates the old cell state and the new cell state is changed from the old cell state. The output gate collects the data and information obtained from the existing LSTM unit, and it is defined as follows:

(11) $$o_{t}=\sigma \left(W\left[x_{t},h_{t-1},C_{t-1}\right]+b_{o}\right)$$

Here, $b_{o}$ is the bias term of the output gate. Finally, the output of the LSTM unit is given as follows:

(12) $$h_{t}=o_{t}.tanh\left(C_{t}\right)$$

Therefore, the structure of the LSTM allows information to circulate between time steps. This creates an internal feedback state that allows the network to understand the concept of time and temporal dynamics in the data. After the LSTM output, two dense layers are connected to the network. The dense layer multiplies the inputs with a weight matrix $w_{y}$ and adds a bias vector $b_{y}$, as below:

(13) $$y_{t}=b_{y}+\sum h_{t}.w_{y}$$

To avoid overfitting, a dropout layer (*D*) is also used between the dense layers. Finally, the softmax layer with the class possibilities is used in the classification stage and it is expressed as follows:

(14) $$S_{k}=\frac{e^{x_{k}}}{\sum_{n=1}^{N}e^{x_{n}}}$$

The output $S_{k}$ is calculated for each input value $x_{k}$, and the sum of all values is equal to 1. The LSTM network is a highly efficient method for sequence data classification due to the possible delay of unknown duration between significant events in the time series. The final network consists of two stacked LSTM layers with 250 hidden units, a dropout layer with a rate of 0.5, dense layers, softmax, and output layer.

### 2.4. Feature Extraction

The statistical approach is one of the effective methods for feature extraction, which achieves great success in the field of time-series data [49]. Also, the size of time-series inputs from different IMU channels (Euler angles) can reach a high size for the network. Therefore, an efficient feature extraction method gives representative information from IMU measurements during complex terrain locomotion with improving model learning, input sizes, and network performance.

The input data consists of yaw (*ψ*), pitch (*θ*), and roll (*ϕ*) angles. For a satisfactory classification performance, it can reflect time, frequency, and spatial feature characteristics. Therefore, the temporal data is divided into segments using a sliding-window and the segmented data are extracted with mean (*m*), median (*med*), LPSD, and WPSD methods. Since the variation of the collected IMU data is time- and amplitude-dependent, these methods enable them to characterize both time-domain and frequency-domain aspects of inertial data collected during robot motion. The *m* and *med* are basic statistical descriptors that represent the central tendency of the signal. The *m* captures the average amplitude, while the *med* provides a measure less affected by noise [50]. Power spectral density (PSD) performs spectral analysis in the frequency-domain, which complements time-domain features in terrain classification. While LPSD provides spectral analysis without requiring equally spaced data points in processing irregular time-series data in different terrains, WPSD increases the reliability of spectral analysis by segmenting the signal and averaging the resulting periodograms [51,52]. In this phase, the feature extraction on a fixed-length sliding-window over time generates distinctive features from the IMU data obtained from the different terrains. This process also reduces the computational cost of the training. As the *m* and *med* define the data distribution for each angle, they can generate meaningful features. The *m* of a window size (*X*) is determined as follows:

(15) $$m=\frac{1}{N}\sum_{i=1}^{N}X_{i}$$

The med is the middle-ranked value of the sorted window and is calculated as follows:

(16) $$med=X_{\left((N+1)/2\right)}\;\;(N\;odd);\;\;\;med=(X_{\left(N/2\right)}+X_{\left(N/2+1\right)})/2\;\;(N\;even)$$

where *X*_(*i*)_ denotes the *it*h order statistic (i.e., the *i*th smallest value) of the samples within the window of length *N*. The spectral representation gives the distinctive features obtained from windowed signals and the LPSD is expressed as follows:

(17) $$LPSD=\frac{1}{2\sigma^{2}}\left[\frac{{\left\{\sum_{k=1}^{N}\left(x_{k}-\bar{x})cos(2\pi f\left(t_{k}-\tau \right)\right)\right\}}^{2}}{\sum_{k=1}^{N}{cos}^{2}(2\pi f\left(t_{k}-\tau \right))}+\frac{{\left\{\sum_{k=1}^{N}\left(x_{k}-\bar{x})sin(2\pi f\left(t_{k}-\tau \right)\right)\right\}}^{2}}{\sum_{k=1}^{N}{sin}^{2}(2\pi f\left(t_{k}-\tau \right))}\right]$$

where $t_{k}$ is the arbitrary time sample with a mean value $\bar{x}$ and a variance $\sigma^{2}$. Here, τ is given as follows:

(18) $$\tau =\frac{1}{2(2\pi f)}arctan\left(\frac{\sum_{k=1}^{N}sin(2\left(2\pi f\right)t_{k})}{\sum_{k=1}^{N}cos(2\left(2\pi f\right)t_{k})}\right)$$

The WPSD is also expressed as follows:

(19) $$WPSD=\frac{1}{X}\sum_{i=1}^{N-1}{\hat{P}}_{i}^{x}$$

**Figure 5:**
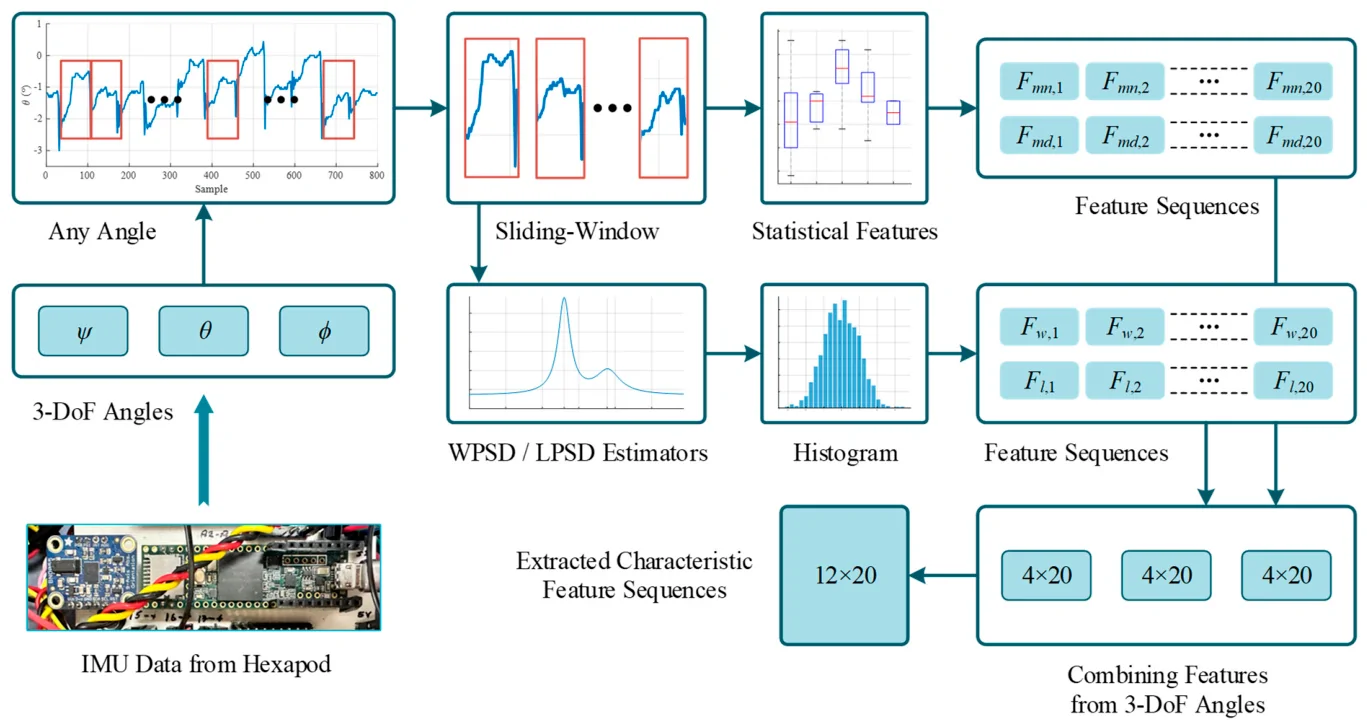
Schematic diagram of the feature extraction process: *ψ*, *θ*, and *ϕ* represent the yaw, pitch, and roll, respectively.

Here, ${\hat{P}}_{i}^{x}$ is the modified periodogram. Finally, the extracted characteristic features are fed to the network input. The feature extraction process is given in Figure 5.

**Figure 6:**
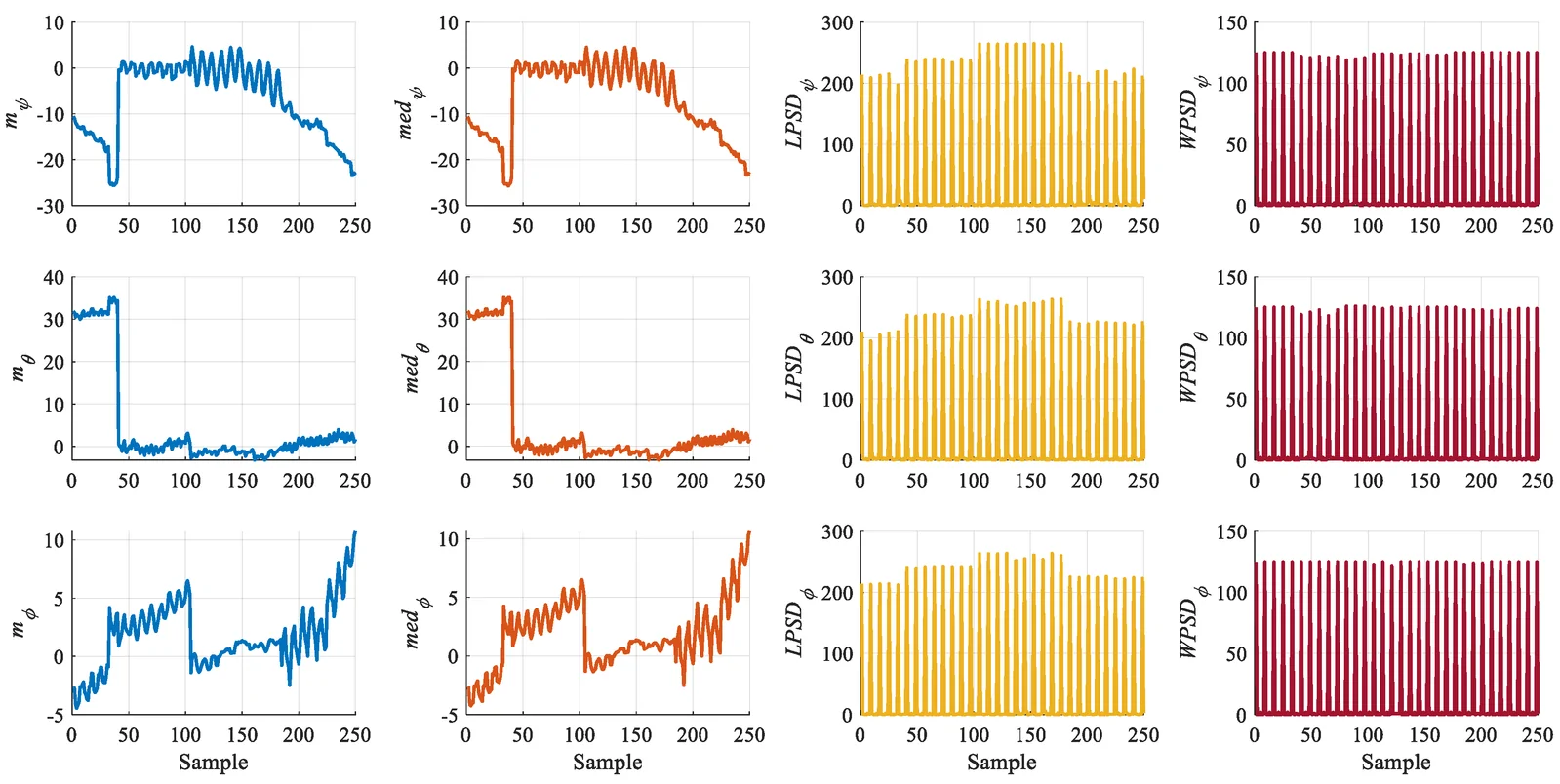
An example of the extracted features from the IMU.

The yaw (*ψ*), pitch (*θ*), and roll (*ϕ*) angles are segmented into fixed-length sliding-windows before feature extraction. The window length *L* is chosen according to the gait segment, and the step size is equal to *L* in all gait samples. Therefore, the window length is directly dependent on the leg period performed during walking and the window length ranges from 110 to 140 samples with non-overlapping windows. For each window and each axis, four feature sets are extracted as m, med, LPSD, and WPSD. Each feature set is represented using a (1 × 20) feature vector, yielding a (4 × 20) feature matrix per axis and (12 × 20) per sample when combining Euler data. Figure 6 presents an example of the extracted features from measurements. For each angle, (4 × 20 × *N*) feature sets are extracted, where *N* is the number of extracted windows. Therefore, the IMU data is constructed as (12 × 20 × *N*) for one terrain. It should be noted that the window length is not tuned manually; it is set to exactly one full leg cycle, *T* = *T^st^* + *T^sw^*, which is analytically determined from the gait parameters. Since the gait patterns have different duty factors at the same CPG frequency of 2 Hz, the cycle duration varies between 110 and 140 samples. In practice, the controller always knows the active gait parameters, and the segmentation is performed automatically from the CPG period without any signal-dependent estimation. The construction of the network input can be summarized as follows. Each fixed-length window, corresponding to one full leg cycle, yields one value per feature and per Euler angle, resulting in a 12-dimensional feature vector (4 features × 3 angles) per window. A total of 20 consecutive windows are then concatenated along the temporal axis to form one input sample. Therefore, each sample is presented to the network as a sequence of 20 time steps, where each time step carries a 12-dimensional feature vector; that is, the input shape is 12 × 20 (feature dimension 12, sequence length 20), and the feature matrix is not flattened.

### 2.5. Data Acquisition

Experiments with the hexapod robot are conducted on 5 different terrain types as concrete, grass, pebble, soil, and waxed tile. While concrete, grass, pebble, and soil represent outdoor environments, waxed tile is selected to represent an indoor environment. Pebble terrain reflects the irregular and unstable surfaces composed of small gravel of varying geometric shapes commonly found in natural outdoor environments. The soil terrain represents a deformable surface containing fine gravel in various areas. In this respect, it is similar to the pebble type of terrain, but the walking patterns are more regular. Grass represents the soft, vegetated ground frequently encountered in natural areas. Concrete is equivalent to hard, uneven, and structured surfaces. Lastly, waxed tile is selected to model smooth and rigid indoor floors. This contrasts with outdoor surfaces, allowing for motion analysis in controlled indoor environments. To prevent redundancy and increase variability, multiple trials are collected on each terrain while the robot performs the four gait patterns as wave, tetrapod, transition, and tripod. Therefore, all combinations of terrain types and gait modes can be obtained. All experiments are conducted under dry surface conditions, and all data are collected on both flat and sloped surfaces to reflect realistic environmental variations and enhance generalization capability. While a terrain represents a flat surface in any environment, it can be on a sloped surface in another environment. Therefore, the collected dataset covers a large number of environment scenarios for the walking of hexapod robots, and signals in each class have varying temporal and spectral properties. It can also be noted that for the pebble-type terrain, only stony areas with geometric shapes that do not hinder the walking of the robot are utilized.

**Figure 7:**
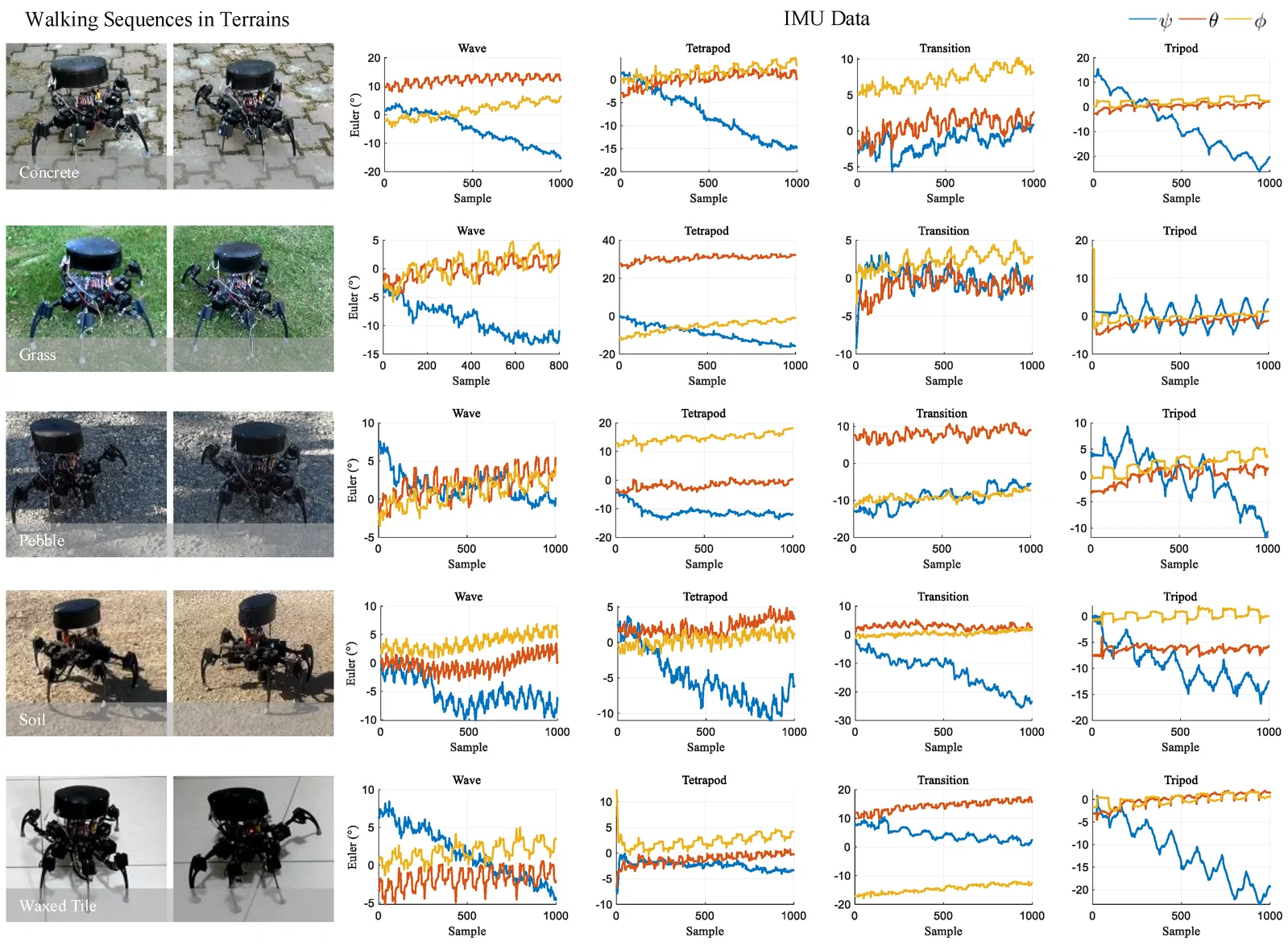
Terrain types and dataset sequences (in degree) obtained from the Euler angles.

The hexapod locomotion is generated by the CPG-based controller, where the CPG outputs drive a trajectory generator and inverse kinematics to produce joint commands. During data collection, the robot walks at a constant frequency of 2 Hz, and gait modes are executed using explicit switching commands. For each trial on a given terrain, IMU data are collected across the four gait patterns as wave, tetrapod, transition, and tripod. The dataset consists of IMU sensor measurements in three angular positions over time. Angular positions in the dataset reflect different situations where the robot encounters one of five commonly seen terrains during navigation. To avoid overfitting in the training for each terrain type, data samples are collected from different parts of the terrain. The data measurements are obtained from the IMU at 100 Hz. All data is recorded in the onboard flash memory and transferred to a computer. For the experiments, the minimum walking time in any environment is set at 10 s. The IMU measurements consist of at least 1000 samples for each angle. The obtained IMU data are segmented into fixed-length windows to form the supervised samples. Using the (12 × 20) sequence data obtained from the feature extraction, a total of 220 packet-windowed data is derived for all terrain types. Walking sequences on the terrains and the corresponding datasets are presented in Figure 7. For clarity, the overall data collection procedure can be summarized as follows: (i) the robot is placed on the selected terrain and walking is started with the wave gait; (ii) the gait is then switched to the tetrapod, transition, and tripod patterns in turn using the explicit switching commands, with at least 10 s of continuous walking per gait at a CPG frequency of 2 Hz; (iii) the three Euler angles are logged at 100 Hz to the onboard flash memory and transferred to the host computer after each trial; (iv) this procedure is repeated on flat and sloped sections and on different areas of each terrain to prevent redundancy and to increase variability; and (v) the recordings are segmented into non-overlapping windows as described in Section 2.6, yielding 220 windowed samples in total for the five terrain classes.

### 2.6. Framework of the Proposed Method

**Figure 8:**
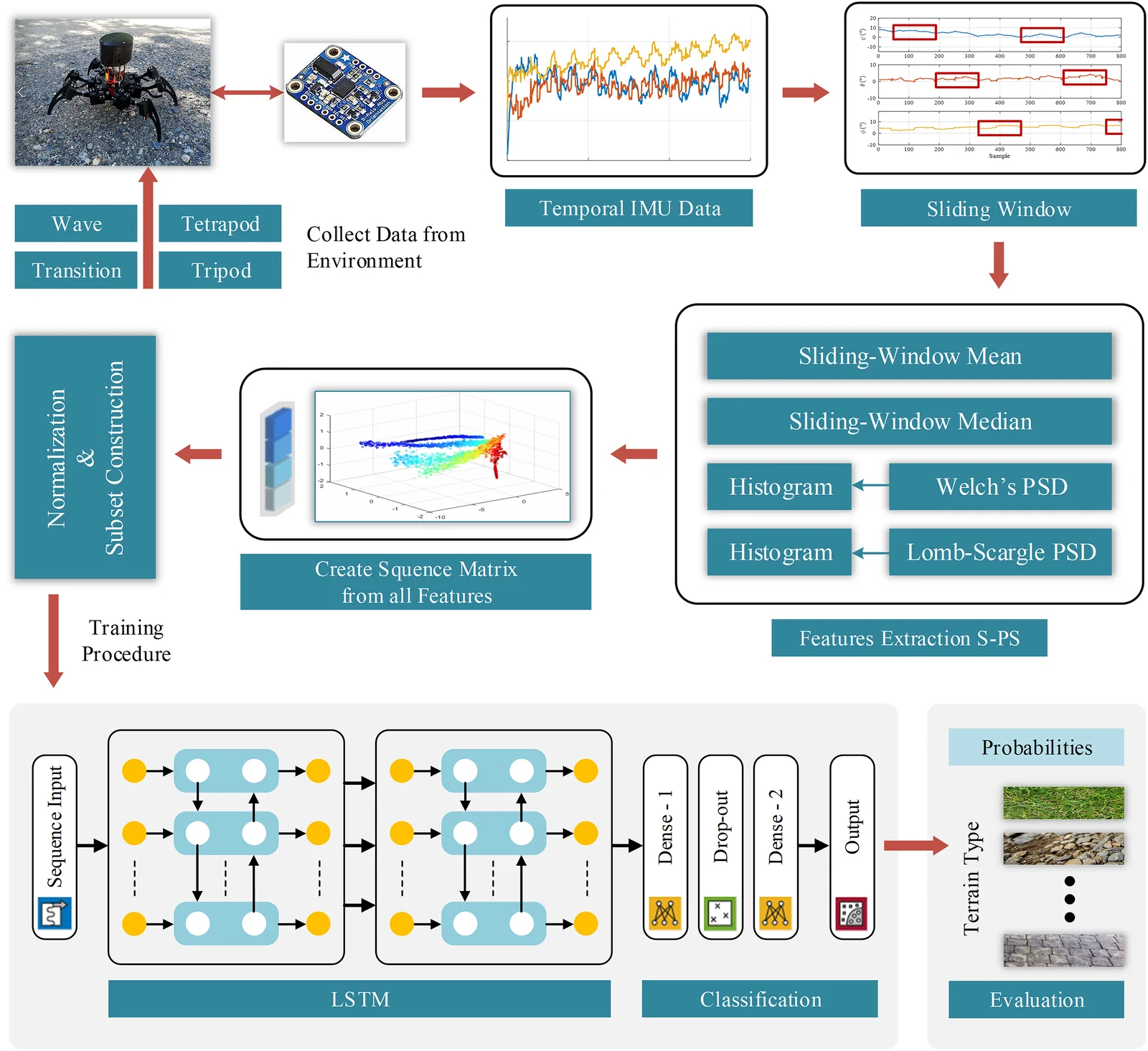
General framework of the proposed method. The pipeline proceeds from data acquisition, preprocessing, and sliding-window segmentation to feature extraction (*m*, *med*, LPSD, and WPSD per Euler angle), sequence construction (12 × 20 feature matrices), and the LSTM-based classification stage with the terrain label output.

The general framework of the proposed terrain classification method is illustrated in Figure 8. The proposed approach combines the statistical and power spectral density (SPSD) feature extraction from the IMU-based angular trajectories with an LSTM network. For clarity, the overall framework will be referred to as SPSD-LSTM throughout the remainder of the paper. The classification structure consists of three main stages as preprocessing, feature extraction, and the LSTM-based deep network model.

In the preprocessing, the yaw angle data collected from the terrain experiments are initially in the range of 0–360 degrees. To ensure correct orientation representation, these values are transformed into the range of ±180 degrees. Undefined data resulting from recording errors is removed from the dataset. For each terrain type, all gait modes are sequentially organized to construct a representative dataset corresponding to that specific terrain. Due to the potential for irregular IMU data between gait transitions, this data is also removed from the dataset. In addition, data recorded during the transition phase between powering the robot and its leg contact with the surface is removed as irrelevant. The feature extraction process provides distinguishing features obtained from the onboard IMU sensor using statistical and PSD methods. The IMU data represents the three-dimensional orientation of the hexapod robot during walking. In the data collection, flat and sloped samples are collected from different parts of an environment to provide comprehensive and realistic terrain data. The Mahony filter is also used to estimate the orientation of a robot using raw data obtained from the IMU. This filter provides a robust and computationally efficient quaternion-based position estimation by combining gyroscope, accelerometer, and magnetometer measurements [53]. The feature extraction process generates the sequence matrix from the obtained features and assists in improving network performance via more appropriate data. After feature extraction, the LSTM network-based classification model is constructed to detect the terrain types. The network architecture comprises two stacked LSTM layers, each followed by a dropout layer to reduce overfitting, and is further connected to dense layers with a softmax and classification output. Each LSTM unit consists of 250 neurons, with a dropout rate set to 0.5. The optimal number of neuron is also determined through a grid search process. In the training, the adaptive moment estimation (Adam) algorithm is also performed. Finally, the proposed model learns multi-level features to classify different terrain types with strong representation capability.

## 3. Experiments

In this section, terrain classification results for the gait adaptation of the hexapod robot are presented in detail. The experiments are conducted in the MATLAB environment on a system equipped with an Intel (R) i7-10750H CPU (2.60 GHz), NVIDIA Quadro P620 GPU, and 16 GB RAM memory. In the experiments, the obtained results are evaluated with commonly preferred classification methods as decision tree (DT), bagged tree (BT), k-nearest neighbors (KNN), SVM, gated recurrent unit (GRU), 1-D CNN-based Inception-Time (IT), and temporal convolutional network (TCN). The collected IMU measurements are segmented into fixed-length, non-overlapping windows (i.e., step size equal to window length). The extracted feature set (4 × 20 × *N*) per angle is then concatenated as (12 × 20 × *N*), and the final feature set is randomly divided into three parts for 70% training, 10% validation, and 20% testing, ensuring that class proportions are preserved and the test set remains fully held out. The z-score normalization is also performed before training. In the training, the mini-batch size is set to 32 and the maximum epoch is preferred as 100. The learning rate is given as 1 × 10^−2^ and reduced by a drop factor of 0.5 after every 40 epochs. The evaluation metrics, obtained results from the experiments, ablation studies, and performance comparison with the current studies are described in the following subsections.

### 3.1. Performance Evaluation Metrics

According to the obtained confusion matrix, accuracy (Acc), precision (Pr), sensitivity (Sn), specificity (Sp), F1-score (F1), and Matthew correlation coefficient (MCC) metrics are used to evaluate the proposed classification method. Acc value indicates the overall capability of the network. While Pr defines the proportion of correct predictions among the samples assigned to a class, Sn (recall) measures the proportion of actual class samples that are correctly identified. F1 is a harmonic average and takes both Pr and Sn. MCC also gives the differences between the actual and predicted classes. These metrics are given as follows:

(20) $$Acc=\frac{TP+TN}{TP+FP+TN+FN}$$

(21) $$Pr=\frac{TP}{TP+FP}$$

(22) $$Sn=\frac{TP}{TP+FN}$$

(23) $$Sp=\frac{TN}{TN+FP}$$

(24) $$F1=\frac{2\times Pr\times Sn}{Pr+Sn}$$

(25) $$MCC=\frac{\left(TP\times TN\right)-\left(FP\times FN\right)}{\sqrt{\left(TP+FP\right)\times \left(TP+FN\right)\times \left(TN+FP\right)\times \left(TN+FN\right)}}$$

Here, TP, FP, TN, and FN are true positive, false positive, true negative, and false negative, respectively.

### 3.2. CPG Network and Feature Extraction Analyses

Table 1 shows the relationship between the duty factor, threshold, and phase difference parameters of the CPG network during walking. Through this relationship, the hexapod robot can walk in different terrains by adopting the gait type. In the typical gait types, the transition between the gaits provides effective locomotion for the fulfillment of assigned tasks. When the hexapod robot encounters flat terrain, it can adopt the tripod gait to achieve the maximum speed. Also, the wave gait is used to walk on complex terrains under stable conditions. Similarly, tetrapod and transition gait types can be selected depending on the terrain structure.

**Figure 9:**
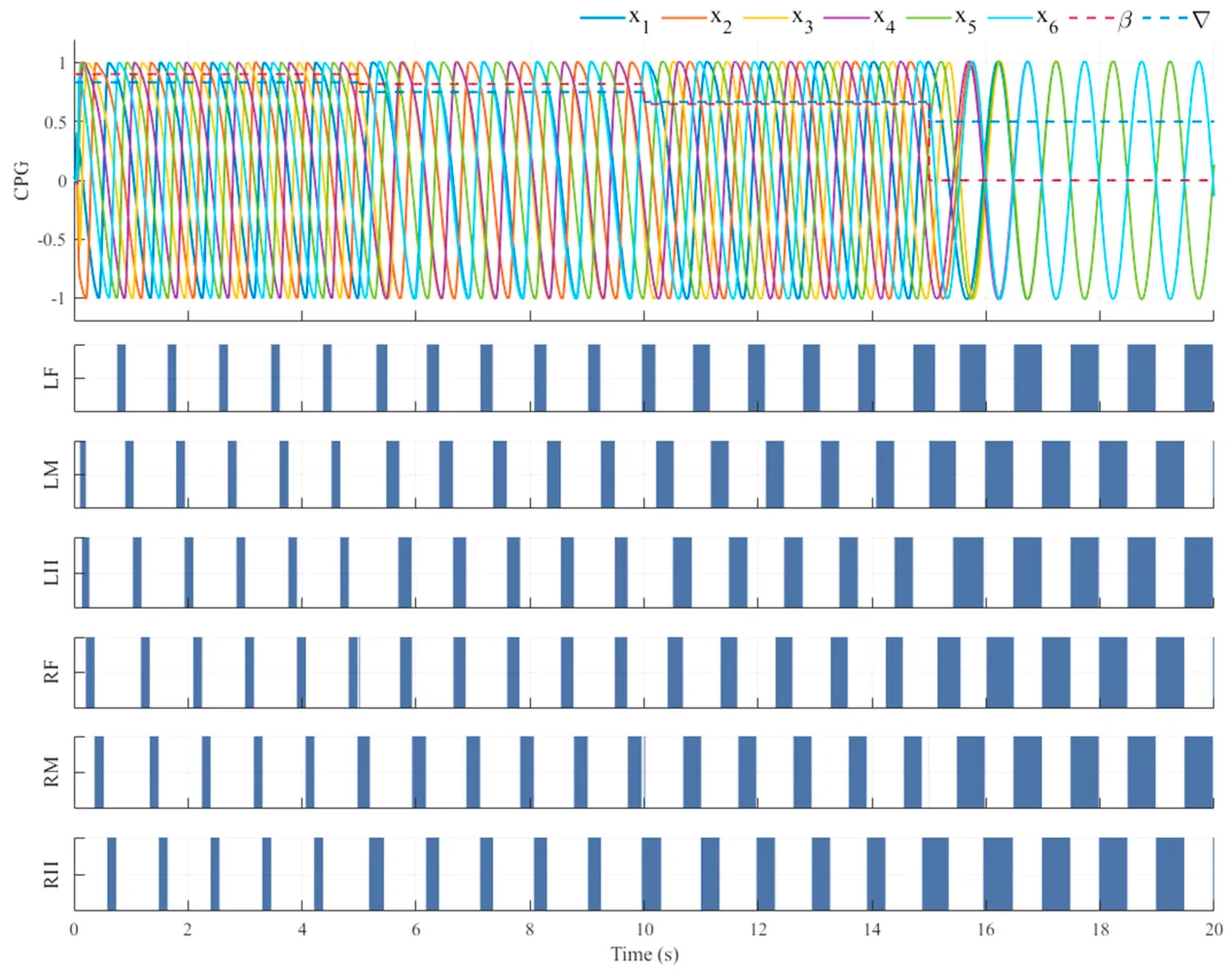
Typical gait diagrams and CPG outputs.

**Figure 10:**
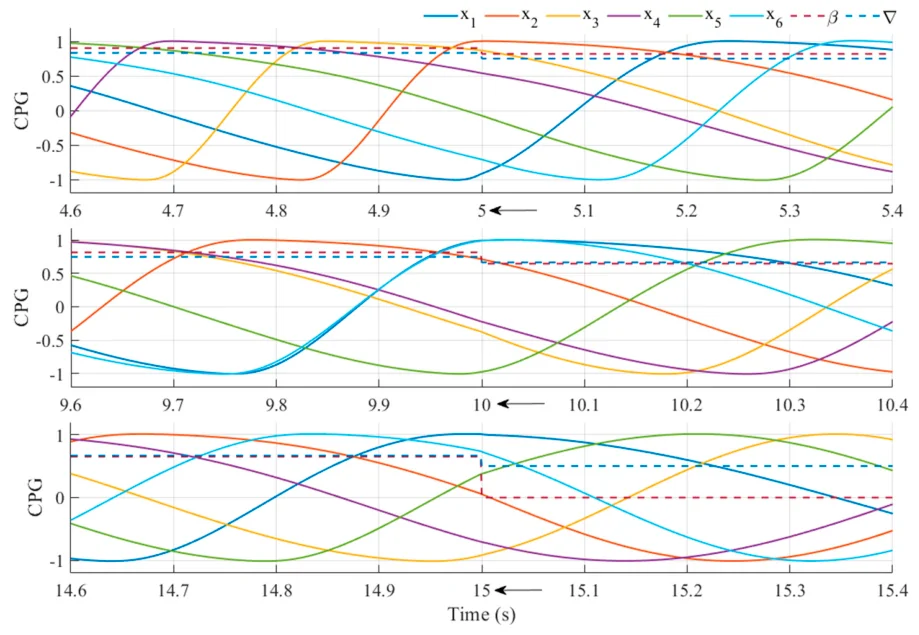
Transition points of the CPG outputs.

Figure 9 presents the CPG network outputs, which generate four typical gait patterns of the hexapod robot. This allows for easy collection of IMU data for all gait modes when a manual trigger signal is sent in any terrain. The robot starts to walk at wave gait and then transverses to tetrapod, transition, and tripod gaits, respectively. The walking speed increases further with each gait transition. For each leg, the blue bars show the swing phase and the white areas show the stance phase. The locomotion of all gaits is performed for 20 s. The transition commands are triggered at *t* = 5 s from wave to tetrapod, *t* = 10 s from tetrapod to transition, and *t* = 15 s from transition to tripod, respectively. The wave gait occurs from front to rear legs as from LF to LM leg, and then LH to RF leg, and finally, RM to RH leg. For this gait, the *β* is selected as 5/6. Similarly, the leg synchronizations are realized for other gaits. Figure 10 also shows the transition points of all gait patterns. These stable and robust phase transitions guarantee smooth and fast gait synchronizations for different terrains. Therefore, the IMU data are collected with all gait types in each terrain types.

**Figure 11:**
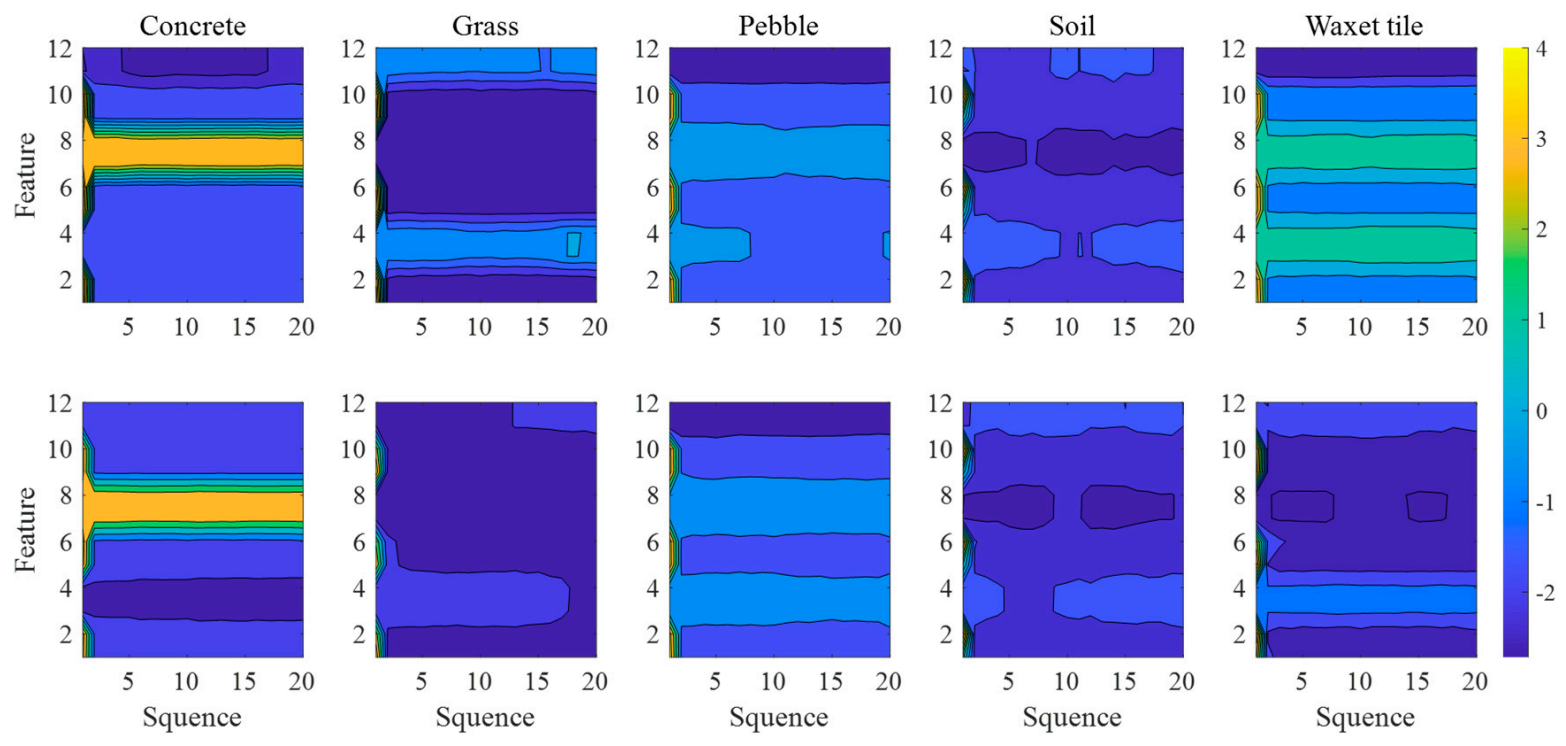
Extracted feature distribution surfaces for all terrain types. The sequence axis denotes the feature vector obtained by time-dependent sliding-window steps; the feature axis represents the combined features (covering LPSD, WPSD, mean, and median features for each row). In each surface plot, the color scale encodes the magnitude of the corresponding feature value.

In Figure 11, the extracted feature distribution surfaces for all terrain types are presented to visualize the distinct topographic structures of each surface. These visualizations illustrate the spatial and spectral characteristics of the robot–terrain interaction. In this representation, the vertical axis corresponds to the extracted features and consists of concatenated features as (4 × 20) per angle derived from the yaw, pitch, and roll angles. This vector includes the LPSD, WPSD, *m*, and *med* values for each Euler angle, respectively. The horizontal axis represents the sliding-window sequence (size of 20), showing the temporal feature vector over the sampling window. Therefore, the size of the feature set equals to (12 × 20). While the features of the pitch angle are more dominant in the concrete terrain, the extracted statistical features of yaw and roll angles are effective in the grass terrain. In the pebble terrain, the yaw and pitch angles are also distinctive. The statistical features of yaw and roll angles are effective for the soil-type terrain. In the waxed tile terrain, it is seen that the statistical features of the yaw angle are effective, while the power statistical features of the roll angle in each sequence are also distinctive. Therefore, it can be seen that Euler angles have a significant representation ability with feature extraction according to terrain types.

### 3.3. Classification Results

The obtained classification results are presented in Table 2. As observed, the proposed SPSD-LSTM outperforms all other methods with an Acc of 95.45%, followed by GRU and TCN with 93.18%, and IT with 88.64%. In contrast, traditional machine learning methods such as DT, BT, KNN, and SVM yield lower Acc values of 81.82%, 81.82%, 79.55%, and 72.73%, respectively. In terms of Pr, the SPSD-LSTM achieves the highest value of 96.36%, while the lowest value is recorded by SVM at 75.98%. GRU and TCN provide relatively close performance with Pr values of 94.36% and 93.86%, whereas IT attains 88.86%. When Sn is examined, the SPSD-LSTM yields the best result at 95.28%, while GRU and TCN follow with 92.78% and 93.06%, respectively; the lowest performance is observed for SVM with 71.94%. When all methods are analyzed in terms of Sp values, the proposed network gives the best value with 98.86%, followed by TCN with 98.30% and GRU with 98.29%, while IT reaches 97.17%. For the F1, the SPSD-LSTM achieves 95.49%, demonstrating a well-balanced trade-off between Pr and Sn. GRU and TCN come next with F1 values of 92.91% and 93.15%, whereas IT records 88.29%, and all other classifiers remain below 82%. In terms of MCC, the SPSD-LSTM provides the highest value of 94.61%, followed by GRU with 91.72% and TCN with 91.70%, while IT achieves 85.72%, and the lowest value is obtained by SVM with 66.63%. From these values, it can be clearly seen that the proposed method outperforms the other methods and makes significant contributions to terrain classification success. With only two incorrect predictions of terrain samples, the proposed method achieves high prediction performance.

To analyze all terrain types, the performance of the proposed SPSD-LSTM is separately evaluated in terms of each class, and the obtained classification results are given in Table 3. According to Table 3, the SPSD-LSTM achieves 100% Acc in the concrete, pebble, and waxed tile classes. Several models also reach the highest Acc in specific terrains; for instance, GRU attains 100% Acc in concrete, soil, and waxed tile, and TCN reaches 100% in concrete and soil, while IT achieves 100% in pebble and waxed tile. For the grass class, the SPSD-LSTM yields 87.50% Acc, which matches the best model performance (TCN at 87.50%) and remains higher than DT, BT, KNN, SVM, GRU, and IT, indicating that grass is among the more challenging classes for most methods. In contrast, the lowest Acc for SPSD-LSTM is observed in the soil class at 88.89%. In terms of Pr, the SPSD-LSTM achieves 100% for concrete, grass, pebble, and soil, while its Pr decreases to 81.82% for waxed tile. A similar trend is observed for IT, where waxed tile Pr is 81.82%, reflecting that non-tile samples can be predicted as waxed tile by some models.

When evaluating Sn, the SPSD-LSTM provides 100% for concrete, pebble, and waxed tile, and it records 87.50% for grass and 88.89% for soil, consistent with the single-sample errors in these two classes. Regarding Sp, the SPSD-LSTM preserves 100% specificity for concrete, grass, pebble, and soil, while waxed tile Sp is 94.29%. When the F1 and MCC are analyzed, SPSD-LSTM attains the strongest results in concrete and pebble, reaching 100% for both F1 and MCC, and it remains competitive in grass and soil. The waxed tile class exhibits comparatively lower F1 and MCC for SPSD-LSTM due to the reduced precision in this class, despite perfect sensitivity. Considering the calculated standard deviations of the SPSD-LSTM performance metrics across terrain classes in Table 3, Acc and Sn show moderate variability with standard deviations of 5.80% each, while Pr varies by 7.27%. Sp exhibits the least variability at 2.28%. The relatively low standard deviations for F1 and MCC, 3.93% and 4.74%, respectively, indicate stable class-wise performance.

**Figure 12:**
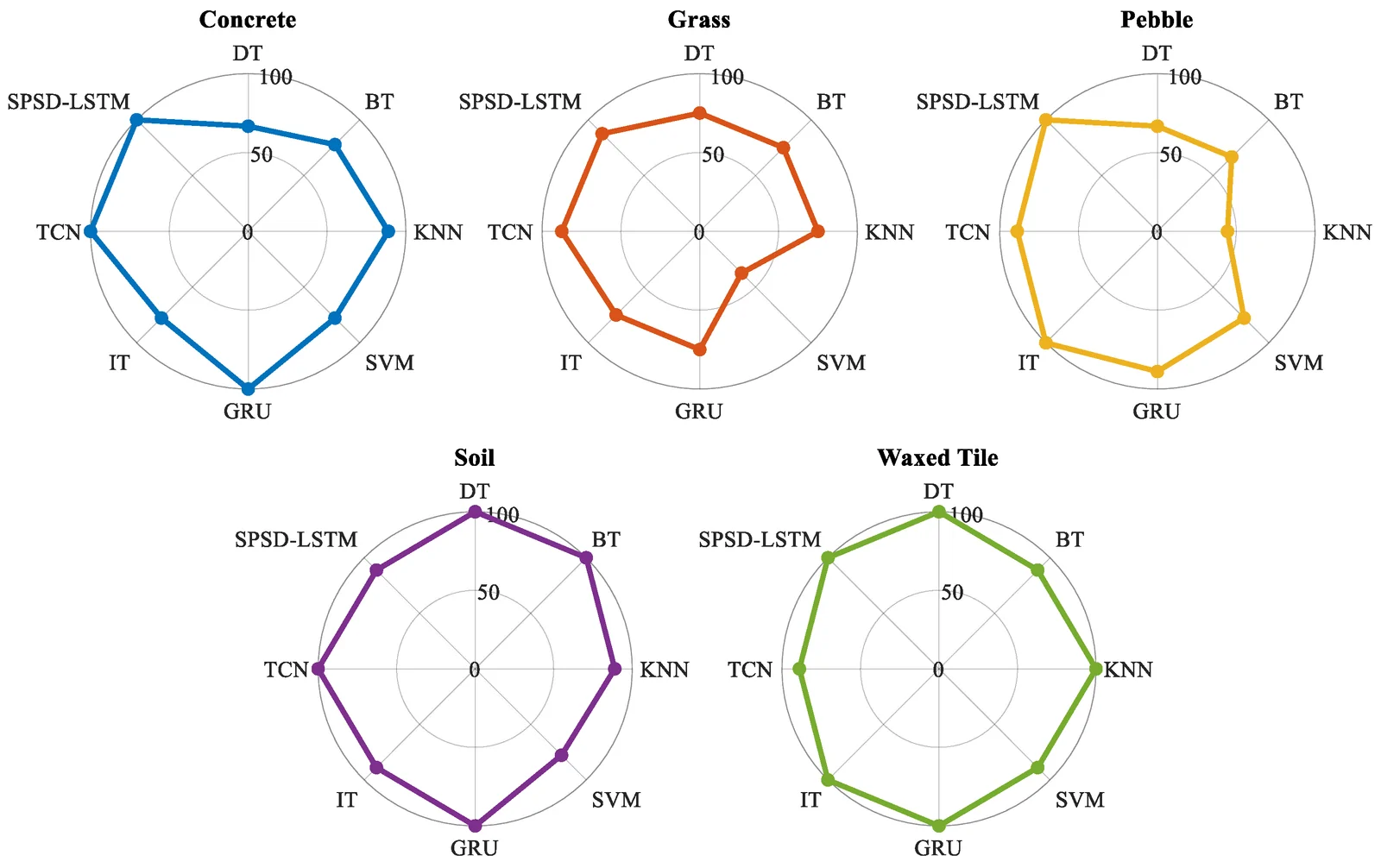
Accuracy comparison of the classification methods according to terrain types.

Figure 12 illustrates the Acc comparison of all classification methods across different terrains. Among these, the grass terrain exhibits the lowest classification performance, whereas the soil and waxed tile terrains achieve the highest Acc values, indicating more distinguishable patterns in the IMU data.

To further illustrate the classification performance of the proposed method, the improvement percentages over the other algorithms are given in Table 4. According to the table, the proposed method achieves notable improvements in classification Acc, particularly over traditional machine learning algorithms. For example, SPSD-LSTM improves the Acc of SVM by 31.24%, demonstrating the highest gain among all methods. Similarly, it improves DT by 16.66%, BT by 16.66%, and KNN by 19.99%, providing consistent performance gains of over 15% among traditional machine learning models. Compared to deep networks, SPSD-LSTM improves IT by 7.68% in Acc, while the smallest Acc improvement is observed for GRU and TCN, both at 2.44%, reflecting their relatively strong baseline performance. The lowest improvement in Pr is observed with GRU at 2.12%, while the highest gain is achieved with SVM at 26.82%; improvements over IT and TCN are 8.44% and 2.66%, respectively. The proposed method also significantly improves Sn, yielding an increase of 32.44% for SVM and 19.94% for KNN, whereas the smallest Sn improvement is observed for TCN at 2.39%. In contrast, the improvements in Sp are relatively lower across all methods, remaining below 6.12%, with the highest rise of 6.12% observed in SVM. The most significant improvements are seen in the F1 and MCC metrics, which reflect balanced classification and overall prediction reliability. F1 shows a 33.16% improvement for SVM and 21.88% for KNN, while MCC improvements reach 41.99% for SVM and 27.01% for KNN. In summary, the proposed method provides higher gains over traditional machine learning algorithms and partially improves deep networks.

**Figure 13:**
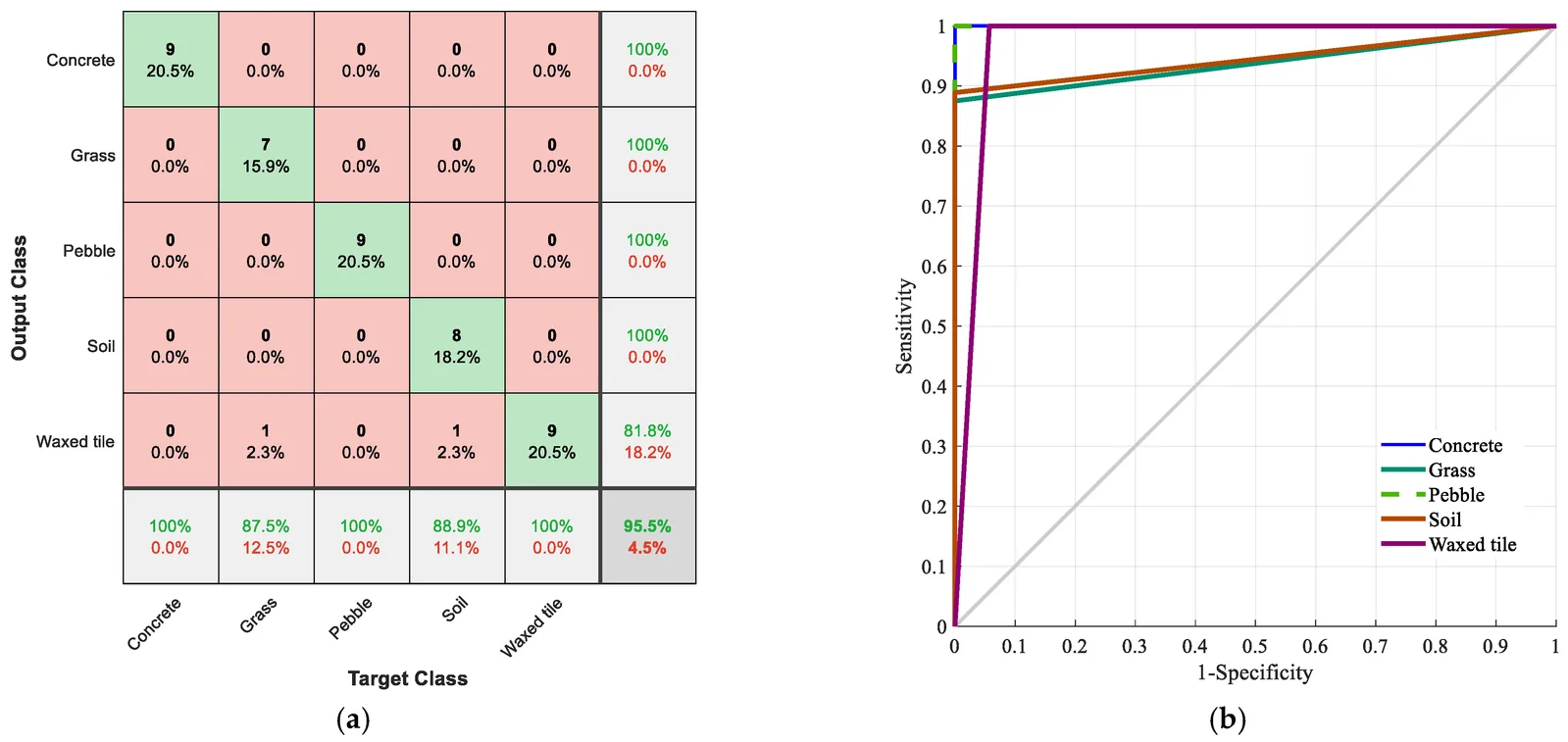
(**a**) Confusion matrix and (**b**) ROC curve of the proposed method.

In Figure 13a, the confusion matrix of the proposed method is presented. According to the confusion matrix, the SPSD-LSTM achieves the highest Sn values for concrete, pebble, and waxed tile, while a single sample is misclassified as waxed tile in both the grass and soil classes. The waxed tile exhibits 100% Sn, but the Pr is 81.82% because two non-tile samples (one grass and one soil) are incorrectly predicted as waxed tile. Since the proposed method relies solely on body orientation, the occurrence of low-excitation dynamics in a window can make the prediction difficult in some terrain samples. Although terrains such as grass and soil have more distinctive surface textures, their deformable structure can reduce high-frequency foot contacts in some surface areas. In addition, the hard and smooth surface of the waxed tile causes a decrease in energy in the PSD features and proximity values in the statistical features during walking. Therefore, angle changes and walking periods with small deviations shift to the waxed tile class in the LSTM network when orientation variability is reduced.

The receiver operating characteristic (ROC) curves can be plotted to more effectively show the performance of the proposed method in classifying terrain types in each class. The ROC curve of the proposed method is shown in Figure 13b and all curves are presented separately for each class. According to the results, the concrete and pebble classes achieve an area under the curve (AUC) value of 1.0, indicating the best classification performance. The waxed tile class also exhibits second-best performance, with an AUC value close to 1. In contrast, the grass and soil demonstrate slightly lower AUC due to the presence of two misclassified samples, which impacts the true positive rate. As a result, these curves demonstrate the high discriminative aspect and high classification capability of the proposed SPSD-LSTM method for the terrain classification using only proprioceptive data.

In Table 5, general comparison of the model complexity is given. The number of learnable parameters (Params), overall memory size (OMS) for the trained network, training time (TrT), and the testing time (TT) are compared in the table. When the number of LSTM units is increased from 1 to 2, the number of learnable parameters increases from 289K to 790K, and the OMS from 1.03 MB to 2.83 MB. Although both parameters increase by almost three times, the model size is still quite small for embedded system applications. Despite increasing model size, TrT decreases from 62 s to 52 s. The TT also shows a significant decrease from 196 ms to 89 ms, indicating that the stacked LSTM configuration not only provides better generalization but also more efficient running time performance. These results show that increasing the number of LSTM units increases the ability of the model to capture temporal dependencies without incurring a commensurate computational cost. Therefore, the applicability of the proposed method is well-suited for real-time terrain classification in embedded systems.

**Figure 14:**
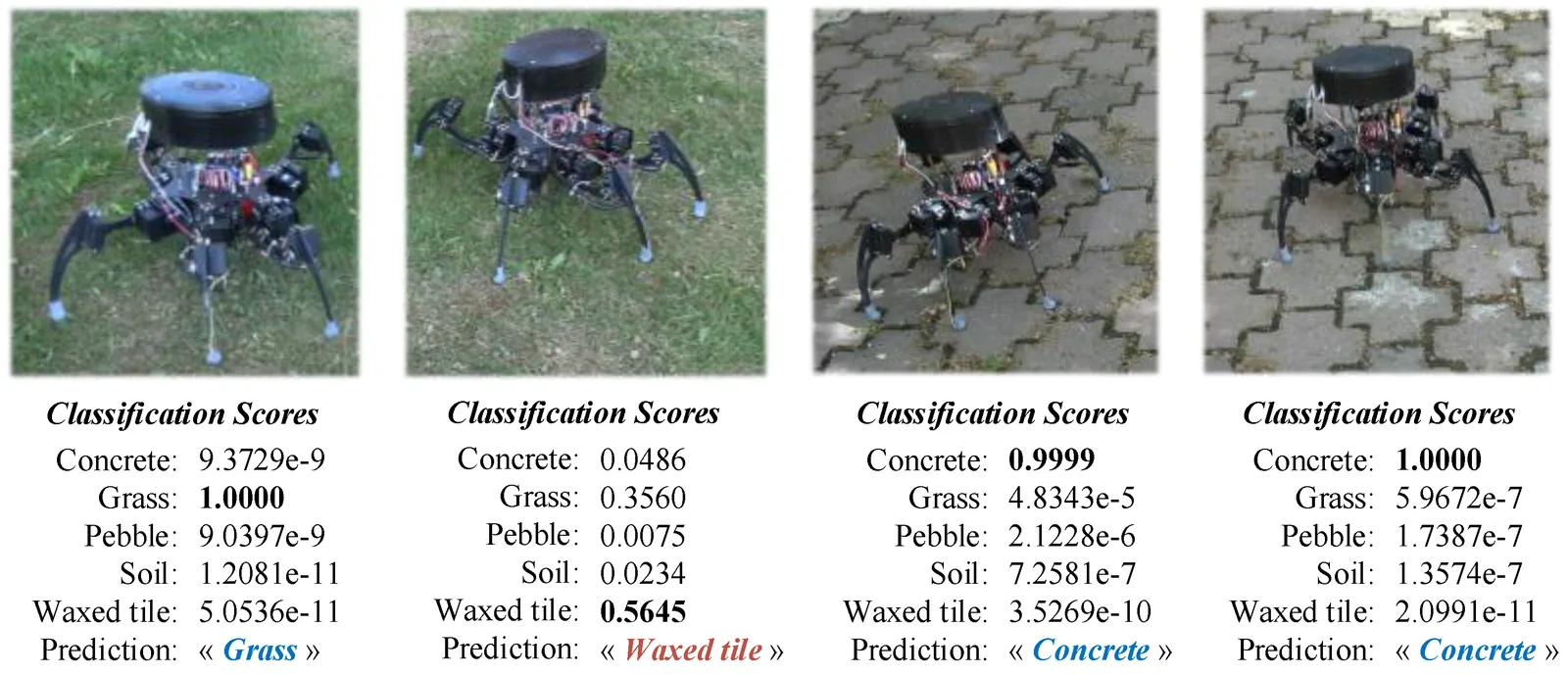
Different terrain classification scores. The blue and red colors represent the correctly and misclassified predictions.

In Figure 14, the classification scores of the proposed method for grass and concrete terrains are given. The hexapod robot walks both terrains at a steady speed for 12 s. For the concrete terrain, the robot achieves the correct classification performance in both start and end positions. These scores are given as 100% at 2 s and 99.99% at 10 s. For the grass-type terrain, the proposed method correctly classifies the terrain at the second gait period, while the sixth gait period is misclassified.

When all classification results are analyzed, the proposed method is suitable for the terrain classification problem and improves the classification on different surfaces. The LSTM network has feedback in the structure, which provides the storage of the necessary information. It can also overcome vanishing gradients and it is capable of handling long-term sequential dependencies. These advantages allow the designed network to give more accurate results in terms of prediction from the IMU data in the experiments. The presented results also demonstrate that the proposed model gives superior performance when it is used with a single proprioceptive IMU sensor. It is also a significant advantage that the proposed method is designed by using the basic biological gait samples of the hexapod robots. Especially, the proposed model has high classification performance without the need for any external sensor and leg design. Therefore, the proposed method can be used in various multi-legged robots embedded with IMU sensors, and successful terrain classification results can be obtained with low cost and are easy to use in real-time applications.

### 3.4. Ablation Studies of the Proposed Method

To evaluate the individual and combined effects of the extracted features on terrain classification performance, an ablation study is conducted using various subsets of four features, namely, LPSD, WPSD, m, and med. Here, PSD denotes the use of the two spectral features (LPSD and WPSD), whereas Statistical refers to the use of the two statistical features (m and med). The notation Dominant: PSD represents combinations containing both spectral features together with one statistical feature, whereas Dominant: Statistical represents combinations containing both statistical features together with one spectral feature. This structured evaluation enables the individual and complementary contributions of the statistical and spectral features to the overall classification performance to be examined.

According to Table 6, the individual use of LPSD, WPSD, and *m* yields an Acc of 20.46%, whereas *med* achieves a higher individual performance of 38.64%, indicating that the median is the most informative single feature among the four considered features. When the features are combined according to their domains, the spectral feature pair (LPSD and WPSD) achieves 40.91% Acc, while the statistical feature pair (*m* and *med*) increases the accuracy to 61.36%. Further combinations provide substantial performance improvements. The combination of WPSD, *m*, and *med*, in which statistical features are dominant, achieves 88.64% Acc. In contrast, the combination of LPSD, WPSD, and *med*, in which spectral features are dominant, reaches 90.91% Acc. The highest classification performance is obtained when all four features are used together, yielding an Acc of 95.45%. These results demonstrate that statistical and spectral features provide complementary information for representing terrain-dependent robot–ground interactions and that their joint use produces the most effective feature representation for terrain classification.

To further investigate the effect of Euler angles, experiments are conducted using different combinations of yaw (*ψ*), pitch (*θ*), and roll (*ϕ*) angles. The number of LSTM units is also varied to observe its impact on performance. The obtained results are summarized in Table 7.

Without feature extraction, using only pitch yields the highest accuracy among single-axis inputs at 59.09%, compared to 56.82% for roll and 45.45% for yaw. Combining multiple angles improves the classification performance, where yaw and pitch reaches 72.73%, and pitch and roll reaches 68.18%. Using all IMU data without feature extraction achieves 70.46% with a one-layer LSTM and 81.82% with a stacked two-layer LSTM. When SPSD feature extraction is applied, performance improves consistently across all configurations. For single-axis inputs, pitch angle reaches 79.55%, which remains higher than roll angle at 65.91% and yaw angle at 61.36%. For two-axis combinations with SPSD features, yaw and pitch reaches 86.36%, yaw and roll reaches 79.55%, and pitch and roll reaches 84.09% using the stacked two-layer LSTM. The best overall accuracy is obtained when SPSD features are extracted from all three angles, reaching 93.18% with a one-layer LSTM and 95.45% with a stacked two-layer LSTM. These results show that the pitch angle is the most informative input, while combining multiple angles further improves classification performance. The slope is distinctive in the classification since it causes changes in body posture due to the direction of locomotion and the load transfer between the front and rear legs during transitions between stance and swing phases. Overall, the ablation results indicate that feature extraction using IMU data significantly improves accuracy, and using two LSTM units provides better performance.

### 3.5. Comparison of the Proposed Method with the State-of-the-Art Studies

In recent years, various multi-legged robots have been developed for different applications and tasks. In this section, terrain classification approaches for the multi-legged robots are presented in detail and classification performances are compared with the proposed method. Table 8 shows the general performance comparison with the state-of-the-art studies.

Wu et al. [9] designed thin and robust capacitive tactile sensors and used them in a small hexapod with C-shaped rotating legs. The sensor measurements included the contact forces of the robot on different types of terrains with high and low friction. The measured parameters were used in the SVM classifier and reached 82.50% of Acc. They also obtained the lowest Acc value for a terrain classification problem of the hexapod robots with the IMU data. Zhang et al. [23] designed a terrain classification model to provide motion planning for a quadruped robot in hard terrain conditions. Labels such as concrete and plastic indicate areas to be flat, while sandy and snowy labels represent slippery surfaces. In their method, IMU and encoder data were combined and an efficient CNN model was used for classification. The overall Acc classification was obtained as 95.02%. Luo et al. [33] used the EfficientNetv2 network as the backbone with an input resolution of 224 × 224. Although the proposed method analyzed a wide range of terrain types, it provided both a high cost and a relatively low Acc value with its perception structure. In another study, Zhu et al. [35] introduced an image infilling method for terrain classification based on a combination of robust features and binary robust invariant scalable key points. The image infilling method was used to identify terrains with obstacles. Finally, the SVM classifier was utilized to classify terrain types. Information on terrains was collected with a Kinect camera. They achieved an average recognition Acc of approximately 85%. Grezmak et al. [36] proposed a terrain classification approach for hexapod robots in which terrain information was collected by measuring component deflection via low-cost hall-effect magnetometers and the IMU. Dataset measurements were collected from three types of granular terrain in both a laboratory and an outdoor environment. They used the SVM classifier and reached an overall Acc of 91.60% with only the IMU and 92.40% with the magnetometer-based leg-compliance sensing for the beach dataset. Zhao et al. [54] proposed a terrain classification method for a hexapod robot. The ground contact force was calculated by collecting joint torques and IMU information. Ground substrates were classified with the extracted feature vector using the SVM algorithm. Three types of terrains were used to collect data as concrete, wood, and foam. They achieved the high Pr and Sn values of above 90% for the three classes and the Acc metric was approximately obtained as 96.67%. Although they achieved the best Acc compared to the proposed method, their terrain class number is less than this study. Also, they combined two types of sensors as force and IMU to collect surface information.

Ahmadi et al. [55] also presented a gated RNN to classify the terrain types of the multi-legged robot. Six types of terrains were analyzed from the public dataset such as concrete, artificial grass, gravel, etc. The mean Acc test result of their method was achieved as 93.20%. In addition, cross-validation leads to confusion about which model to use in embedded system applications, because each obtained model gives a different test performance. It should be noted that the studies summarized in Table 8 employ different robots, terrain datasets, sensing modalities, numbers of terrain classes, and evaluation protocols. Therefore, the given accuracy values should be interpreted as contextual comparisons rather than direct benchmarks. Within this context, the proposed method achieves competitive classification performance while relying on only a single body-mounted IMU sensor and distinguishing five terrain classes. While Zhao et al. [54] achieved a 96.67% prediction accuracy, which is 1.27% higher than the proposed method, these results were achieved with only three classes and a combination of force and IMU sensors. Compared with previous studies using additional sensing or fewer classes, the proposed method maintains competitive performance with only a single IMU sensor and a larger terrain set.

### 3.6. Discussion and Limitations

The proposed SPSD-LSTM framework represents an effective classification performance in distinguishing varying terrain dynamics by combining statistical and spectral features with a deep temporal learning architecture. The experimental results indicate that the fusion of time-domain descriptors with frequency-domain features derived from Euler angles is critical for capturing the kinematics of robot–terrain interaction. While statistical features provide information on the signal amplitude distribution related to surface roughness and deformability (e.g., distinguishing soil from pebble), the spectral power density features encode the periodic structural vibrations occurring on rigid surfaces like concrete and waxed tile. The terrains with 100% performance exhibit distinct interactions in the IMU orientation dynamics. The pebble generates high-frequency excitation due to irregular contacts, while waxed tile produces more regular and low-variance patterns on a rigid and smooth surface. These differences are well captured by the statistical and spectral feature representation and the temporal modeling capability of the LSTM. The LSTM network effectively learns the long-term temporal dependencies from these features, resulting in a high classification accuracy of 95.45% across the five terrain types. Compared to other methods, the proposed approach achieves the highest overall performance and provides great improvements, especially against machine learning models. Although the improvements are limited compared to GRU and TCN, feature extraction-based temporal modeling gives effective classification performance. As quantitatively demonstrated in the results, the performance improvements of the proposed SPSD-LSTM arise from the complementary contributions of statistical and spectral features together with multi-axis temporal information. Furthermore, while axis-based analysis gives the pitch angle as the most informative component, the performance of multi-axis fusion demonstrates that terrain identification can be easily obtained by coupled body oscillations. For embedded systems, the proposed model remains lightweight enough for real-world operations. The model complexity analysis indicates that moving from a single-layer to a stacked two-layer LSTM increases the number of parameters and the memory size, but the overall model size remains small and practical for constrained platforms. Moreover, the testing time is on the order of 10^−1^ s and decreases from 196 ms to 89 ms with the stacked configuration, which supports real-time applications. However, real-time implementation depends on the sampling rate and windowing size used onboard systems, and these parameters and delay intervals should be considered for a specific platform.

Despite the high performance mentioned above, the proposed model has some limitations that need to be considered for real-world applications. While the results demonstrate high performance within the identified classes, the generalizability of the model to different or previously unseen terrains remains an important issue. The current formulation is a closed-set supervised recognition problem, and this model is trained to distinguish between five predefined terrain classes using SPSD feature sequences extracted from IMU, and the generalization ability depends on the similarity of the different terrains to those included in the training set. Consequently, when the robot encounters an unseen surface (e.g., sand, mud, or a different terrain morphology), the representation of this interaction is assigned to one of the learned classes based on the nearest region of the learned decision space, producing an incorrect label. This is a consequence of performing classification without an explicit unknown class. Expanding the training dataset to include a wider range of terrain and environmental conditions will increase the model’s applicability to real-world scenarios, particularly walking in diverse and unpredictable environments. Secondly, while the defined statistical and spectral features provide compact and distinctive information about the robot–terrain interaction, these features are extracted from the body orientation only. Therefore, terrains exhibiting similar dynamics under specific operating conditions may remain partially uncertain in the feature space, and texturally different but dynamically similar interactions can occur. To address these limitations, an unknown terrain class can be added to SPSD-LSTM. Through training with different walking samples from complex terrains, the robot can be guided to a reliable gait mode using the unknown class. Additionally, using an encoder integrated with the LSTM classifier can also define boundary conditions for unseen terrain types. In such a case, it may also be possible to increase the classification performance by obtaining more comprehensive features in feature extraction to evaluate the terrain interaction. In the feature extraction process, incorporating multi-resolution or adaptive features based on speed and gait variations may enable the classifier to better distinguish terrain-dependent dynamics. A further limitation is related to the operating and environmental conditions used during data acquisition. All experiments were conducted at a fixed CPG frequency of 2 Hz and under dry-surface conditions. Consequently, the current results do not directly establish the robustness of the proposed feature representation to changes in walking frequency or to wet, slippery, or otherwise altered terrain conditions. Since variations in gait frequency may modify the temporal and spectral characteristics of the body-orientation signals, the performance of the classifier should be further evaluated over a wider range of locomotion speeds and environmental conditions. Future work will evaluate the proposed method at multiple gait frequencies and under more challenging surface conditions, including wet and highly deformable terrains.

Another limitation concerns the size of the dataset and the evaluation protocol. The obtained results are based on 220 windowed samples and a single data split, which corresponds to approximately 44 test samples. Although the class proportions are preserved, the test set is fully held out, and all compared methods are evaluated under an identical protocol, the absolute accuracy values should be interpreted with the uncertainty of this limited test set in mind. The data collection activities on the hexapod platform are continuing within the scope of the supporting project; a cross-validation analysis on the enlarged dataset, with trial-level separation to avoid temporally correlated windows, is planned as future work to further establish the statistical reliability of the results. Finally, it should be emphasized that the present study focuses on the perception side of adaptive locomotion: the proposed classifier provides the terrain information required for gait adaptation, but classifier-driven gait switching is not demonstrated in this paper. Closing the loop requires additional mechanisms beyond classification accuracy, in particular a decision-making on the classifier output to avoid spurious gait switches and a stability analysis of CPG transitions triggered at arbitrary phases of the gait cycle. The next step of this research is an online closed-loop experiment to trigger autonomous gait transitions across terrains, together with the deployment and onboard timing benchmarking of the network on the microcontroller.

## 4. Conclusions

In this paper, an efficient terrain classification approach using a low-weight and low-cost system is presented. To enable robot–terrain interaction, only a 9-axis proprioceptive IMU sensor is used as the sensing method, and Euler angle measurements of the hexapod robot, which are already available in multi-legged robots and without the need for any external contact sensors, are considered basic surface information. The proposed method combines a deep classification framework including the LSTM network and an effective feature extraction process. In the feature extraction, the four meaningful characteristic features as mean, median, LPSD, and WPSD are obtained from the measurements using the sliding-window method. The extracted features are then constructed as the subset feature vector and fed into the input of the designed LSTM network. Therefore, the interaction of the robot in different environments is obtained as temporal patterns from dynamic data. In robot locomotion, a coupled CPG network-based control architecture is used to generate robust and stable gait patterns. In the experiments, the proposed method is evaluated with commonly preferred classifiers. The IMU data is collected with the wave, tetrapod, transition, and tripod gait types in each terrain to increase the generalization capability of the network. When the quantitative metric results of the proposed method are evaluated, the accuracy values of the proposed method are obtained as 100% for concrete, 87.50% for grass, 100% for pebble, 88.89% for soil, and 100% for waxed tile. The Pr, Sn, Sp, F1, and MCC values of the proposed deep network are calculated as 96.36%, 95.28%, 98.86%, 95.49%, and 94.61%, respectively. The obtained classification results show that the proposed method can not only correctly detect and classify the terrain types but also can play an important role in the design of motion planning algorithms with its easy applicability. Future work will proceed in four directions: (i) an online closed-loop experiment in which the classifier output is fed back to the CPG controller to trigger autonomous gait transitions, together with the onboard deployment and timing benchmarking of the network on the microcontroller; (ii) a cross-validation analysis on an enlarged dataset with trial-level separation; (iii) the use of additional IMU channels, such as angular velocity and linear acceleration, and richer statistical features to extend the perception capability; and (iv) the classification of more diverse and harsh terrain types, such as sand, wet surfaces, and mud-like soil, including an explicit unknown-terrain class.

## Figures and Tables

**Table 1:** Relationship between the duty factor, threshold, and phase difference.

Gait Type	$\emptyset_{ij}$	β	∇
Wave	π/3	5/6	0.8660
Tetrapod	π/2	3/4	0.7071
Transition	2π/3	2/3	0.5000
Tripod	π	1/2	0.0000

**Table 2:** Classification results of all methods (%).

Method	Acc	Pr	Sn	Sp	F1	MCC
DT	81.82	85.85	81.67	95.49	81.70	78.77
BT	81.82	82.24	81.67	95.44	81.67	77.36
KNN	79.55	80.33	79.44	94.90	78.35	74.49
SVM	72.73	75.98	71.94	93.16	71.71	66.63
GRU	93.18	94.36	92.78	98.29	92.91	91.72
IT	88.64	88.86	88.33	97.17	88.29	85.72
TCN	93.18	93.86	93.06	98.30	93.15	91.70
SPSD-LSTM	95.45	96.36	95.28	98.86	95.49	94.61

**Table 3:** Classification results of all methods for each terrain type (%).

Method	Class	Acc	Pr	Sn	Sp	F1	MCC
DT	Concrete	66.67	100.00	66.67	100.00	80.00	78.36
	Grass	75.00	60.00	75.00	88.89	66.67	58.80
	Pebble	66.67	100.00	66.67	100.00	80.00	78.36
	Soil	100.00	100.00	100.00	100.00	100.00	100.00
	Waxed Tile	100.00	69.23	100.00	88.57	81.82	78.31
BT	Concrete	77.78	77.78	77.78	94.29	77.78	72.06
	Grass	75.00	85.71	75.00	97.22	80.00	76.16
	Pebble	66.67	75.00	66.67	94.29	70.59	63.75
	Soil	100.00	100.00	100.00	100.00	100.00	100.00
	Waxed Tile	88.89	72.73	88.89	91.43	80.00	74.82
KNN	Concrete	88.89	80.00	88.89	94.29	84.21	80.06
	Grass	75.00	66.67	75.00	91.67	70.59	63.75
	Pebble	44.44	80.00	44.44	97.14	57.14	52.86
	Soil	88.89	100.00	88.89	100.00	94.12	92.96
	Waxed Tile	100.00	75.00	100.00	91.43	95.71	82.81
SVM	Concrete	77.78	70.00	77.78	91.43	73.68	66.61
	Grass	37.50	75.00	37.50	97.22	50.00	46.59
	Pebble	77.78	77.78	77.78	94.29	77.78	72.06
	Soil	77.78	100.00	77.78	100.00	87.50	85.78
	Waxed Tile	88.89	57.14	88.89	82.86	69.57	62.13
GRU	Concrete	100.00	81.82	100.00	94.29	90.00	87.83
	Grass	75.00	100.00	75.00	100.00	85.71	94.29
	Pebble	88.89	100.00	88.89	100.00	94.12	92.96
	Soil	100.00	100.00	100.00	100.00	100.00	100.00
	Waxed Tile	100.00	90.00	100.00	97.14	94.74	93.50
IT	Concrete	77.78	87.50	77.78	97.14	82.35	78.35
	Grass	75.00	75.00	75.00	94.44	75.00	69.44
	Pebble	100.00	100.00	100.00	100.00	100.00	100.00
	Soil	88.89	100.00	88.89	100.00	94.12	92.96
	Waxed Tile	100.00	81.82	100.00	94.29	90.00	87.83
TCN	Concrete	100.00	81.82	100.00	94.29	90.00	87.83
	Grass	87.50	87.50	87.50	97.22	87.50	84.72
	Pebble	88.89	100.00	88.89	100.00	94.12	92.96
	Soil	100.00	100.00	100.00	100.00	100.00	100.00
	Waxed Tile	88.89	100.00	88.89	100.00	94.12	92.96
SPSD-LSTM	Concrete	100.00	100.00	100.00	100.00	100.00	100.00
	Grass	87.50	100.00	87.50	100.00	93.33	92.27
	Pebble	100.00	100.00	100.00	100.00	100.00	100.00
	Soil	88.89	100.00	88.89	100.00	94.12	92.96
	Waxed Tile	100.00	81.82	100.00	94.29	90.00	87.83

**Table 4:** Improvement performance of the SPSD-LSTM (%).

Method	Acc	Pr	Sn	Sp	F1	MCC
DT	16.66	12.24	16.66	3.53	16.88	20.11
BT	16.66	17.17	16.66	3.58	16.92	22.30
KNN	19.99	19.96	19.94	4.17	21.88	27.01
SVM	31.24	26.82	32.44	6.12	33.16	41.99
GRU	2.44	2.12	2.69	0.58	2.78	3.15
IT	7.68	8.44	7.87	1.74	8.15	10.37
TCN	2.44	2.66	2.39	0.57	2.51	3.17

**Table 5:** General comparison of the model complexity. The training (TrT) and testing (TT) times are measured on the workstation described in Section 3.

Num. of Unit	Num. of Param (K)	OMS (MB)	TrT (s)	TT (ms)
1	289	1.03	62	196
2	790	2.83	52	89

**Table 6:** Ablation results according to feature variations.

Group	Features				Acc (%)
	LPSD	WPSD	*m*	*med*	
Single	√	✗	✗	✗	20.46
	✗	√	✗	✗	20.46
	✗	✗	√	✗	20.46
	✗	✗	✗	√	38.64
PSD	√	√	✗	✗	40.91
Statistical	✗	✗	√	√	61.36
Dominant: Statistical	✗	√	√	√	88.64
Dominant: PSD	√	√	✗	√	90.91
All	√	√	√	√	95.45

**Table 7:** Ablation results according to the angle variations.

Inputs				Num. of Unit	Acc (%)
SPSD	ψ	θ	ϕ		
✗	√	✗	✗	2	45.45
	✗	√	✗		59.09
	✗	✗	√		56.82
	√	√	✗		72.73
	√	✗	√		61.36
	✗	√	√		68.18
	√	√	√	1	70.46
	√	√	√	2	81.82
√	√	✗	✗	2	61.36
	✗	√	✗		79.55
	✗	✗	√		65.91
	√	√	✗		86.36
	√	✗	√		79.55
	✗	√	√		84.09
	√	√	√	1	93.18
	√	√	√	2	95.45

**Table 8:** Performance comparison of the proposed method with the current studies.

Study	Prototype	Method	Sensor	Terrain Class	Acc (%)
Wu et al. [9]	C-Legged Hexapod	SVM	Only Force	4	82.50
			Only IMU		71.50
Zhang et al. [23]	Quadruped	CNN	IMU + Encoder	4	95.02
Luo et al. [33]	Quadruped	EfficientNetv2	Camera	15	90.80
Zhu et al. [35]	Hexapod	Image Infilling + SVM	Camera	6	85.00
Grezmak et al. [36]	Hexapod	SVM	Magnetometer + IMU	3	92.40
Zhao et al. [54]	Hexapod	SVM	Force + IMU	3	96.67
Ahmadi et al. [55]	Quadruped	RNN + FCL	Force + IMU	6	93.20
This Paper	Hexapod	SPSD-LSTM	Only IMU	5	95.45

## Data Availability

The dataset used in this study contains only robotic proprioceptive IMU measurements. Due to ongoing project activities, the dataset and the associated code will be available upon request.
